# Bonding Situation of σ‐E−H Complexes in Transition Metal and Main Group Compounds

**DOI:** 10.1002/chem.202201920

**Published:** 2022-08-22

**Authors:** Pablo Ríos, Salvador Conejero, Israel Fernández

**Affiliations:** ^1^ Instituto de Investigaciones Químicas (IIQ) Departamento de Química Inorgánica Centro de Innovación en Química Avanzada (ORFEO-CINQA) CSIC and Universidad de Sevilla Avda. Américo Vespucio 49 41092 Sevilla Spain; ^2^ Departamento de Química Orgánica Centro de Innovación en Química Avanzada (ORFEO-CINQA) Facultad de Ciencias Químicas Universidad Complutense de Madrid Cuidad Universitaria 28040 -Madrid Spain

**Keywords:** σ-SiH complexes, backdonation, bonding situation, DFT calculations, Energy Decomposition Analysis

## Abstract

The ambiguous bonding situation of σ‐E−H (E=Si, B) complexes in transition metal compounds has been rationalized by means of Density Functional Theory calculations. To this end, the combination of the Energy Decomposition Analysis (EDA) method and its Natural Orbital for Chemical Valance (NOCV) extension has been applied to representative complexes described in the literature where the possible η^1^ versus η^2^ coordination mode is not unambiguously defined. Our quantitative analyses, which complement previous data based on the application of the Quantum Theory of Atoms in Molecules (QTAIM) approach, indicate that there exists a continuum between genuine η^1^ and η^2^ modes depending mainly on the strength of the backdonation. Finally, we also applied this EDA‐NOCV approach to related main‐group species where the backdonation is minimal.

## Graphical abstract

**The controversial bonding** situation of σ‐E−H (E=Si, B) complexes in transition metal and main‐group compounds is quantitatively analyzed by means of computational tools. This study sheds light on the crucial role of backdonation to determine the possible η^1^ or η^2^ coordination modes.

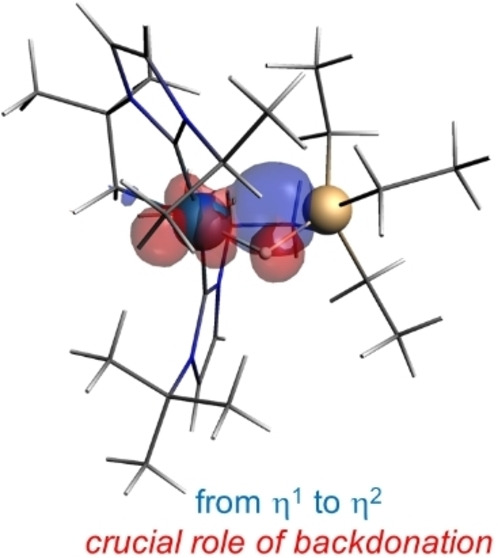

## Introduction

**Figure 1:**
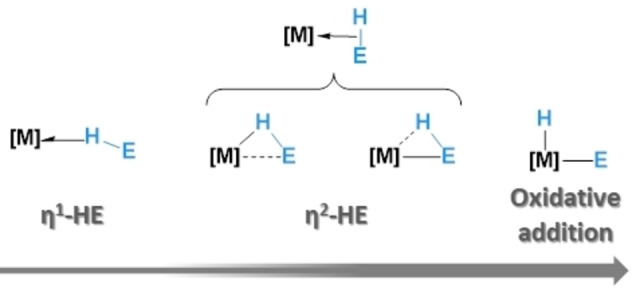
Possible coordination modes in σ‐E−H complexes.

The nature of the interaction of E−H bonds (E=B, Si, etc) with transition metals and some main group elements (to form σ‐EH complexes) is key to understanding their reactivity and stability. Since the seminal report by Graham on the first σ‐SiH complex,^[1]^ fueled by the discovery by Kubas of a σ‐H_2_ compounds,^[2]^ there has been an increasing number of this type of compounds that have been isolated and characterized by X‐ray and neutron diffraction studies.^[3]^ More recently, the renaissance of the chemistry of main group elements, particularly those of the *p*‐block, has uncovered the ability of some Lewis acids to interact with hydrosilanes to form the corresponding σ‐SiH complexes.^[4]^ This has led to the discovery of new reactivity patterns involving the formal transfer of silylium cations to organic molecules, which has also been observed on some occasions with transition metals.^[5]^ Depending on the nature of the Lewis acid (i.e., transition metal or main group element), several bonding scenarios can be envisaged. Regarding, for example, hydrosilanes and transition metals, the polarity of the Si−H bond develops an unsymmetrical interaction with the metal center that can be viewed as a continuum en route to the cleavage of the Si−H bond (oxidative addition) in which the starting point constitutes the interaction through the H atom (end‐on coordination, or η^1^) (Figure 1).

**Figure 2:**
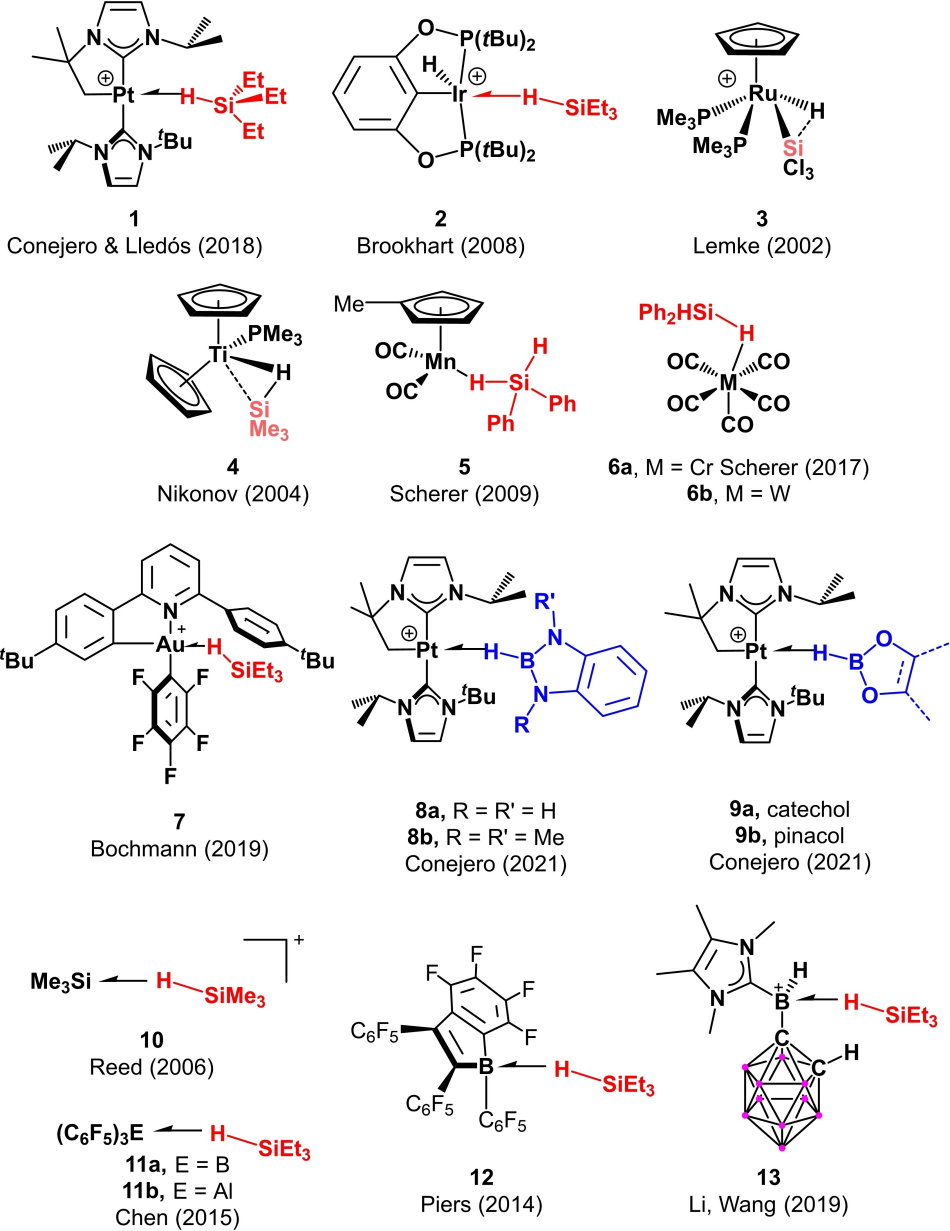
Representative compounds studied herein.

Subsequently, as reported by Scherer,^[6]^ the system can evolve through an intermediate η^2^ coordination (through both the hydrogen and silicon atoms) whose metrical parameters are mainly governed by the ability of the metal to back‐donate into the σ*(SiH) orbital, and steric factors.[^3^, ^7^] If backdonation is sufficiently strong, complete cleavage of the Si−H bond can take place. *p*‐Block σ‐SiH complexes have been reported to exhibit an η^1^ coordination,[^4b^, ^4c^, ^4d^] with one exception,^[4a]^ since their ability to back‐donate is typically negligible. The opposite is usually observed in transition metal complexes, for which in most cases the η^2^ is prevalent. However, the extent of the interaction of the metal and silicon atoms in this latter bonding scenario can be, sometimes, difficult to characterize. As stated by Nikonov in 2005, “…*this designation (*η^2^) *does not tell us anything about the origin of the interaction and is entirely ambiguous”*.^[3e]^ In a recent contribution by our group, we synthesized and spectroscopically observed some σ‐SiH cationic Pt(II) complexes of the formula [Pt(NHC’)(NHC)(HSiR_3_][BAr^F^], one of which was characterized by X‐ray diffraction studies.^[8]^ The metrical parameters did not allow us to clearly distinguish the type of interaction, and NMR data were identical to those reported for the only transition metal‐based σ‐SiH complex reported to have an η^1^ binding mode.^[7]^ Interestingly, the application of Atoms‐in‐Molecules (QTAIM) methods on our platinum systems indicated the absence of either bond paths (BPs) or bond critical points (BCPs) between the platinum and silicon atoms, suggesting coordination only through the hydrogen atom. However, this system, and some others, are thermally unstable and evolve through Si−H bond cleavage, indicating that, at some point, an interaction between these two atoms is necessarily forged (i.e., η^2^ interaction). In addition, the calculated energy for bending or widening the Pt−H−Si angle proved to be quite low for arranging an almost linear η^1^ type interaction.^[8]^ On the other hand, complexes Cr(CO)_5_(HSiHPh_2_)^[9]^ and Mn(Cp’)(CO)_2_(HSiHPh_2_)^[10]^ have been reported as η^1^ and η^2^ derivatives, respectively, despite the lack of BCPs between the metal and silicon atoms in both complexes.^[11]^ The η^2^ assignment in the manganese case was based on the observed relatively short Mn⋅⋅⋅Si distance and the higher and lower electron density at the Mn−H and Si−H BCPs, respectively.^[11]^ Therefore, the boundaries between η^1^ and η^2^ coordination modes appear to be very thin and difficult to discern. Thus, it becomes evident that the application of AIM methods alone does not provide a clear‐cut rationalization of the bonding situation in these species. In order to have a better understanding and a more accurate picture of the nature of this interaction, reliable and complementary approaches should be used instead to quantify the extent of the potential M⋅⋅⋅Si interaction. In this contribution, we analyze the interaction of hydrosilanes (and some boranes) with metal complexes and *p*‐block‐based compounds by means of state‐of‐the‐art computational methods in bonding analyses, namely the Energy Decomposition Analysis^[12]^‐Natural Orbital for Chemical Valence^[13]^ (EDA‐NOCV) method in combination with the Natural Bond Orbital^[14]^ method. In particular, the former approach (EDA‐NOCV) has been chosen because it has been proven to provide reliable and quantitative insight into the bonding situation in both transition metal complexes and main‐group compounds.^[15]^ These calculations will allow us to provide a more realistic description of the interaction present in a number of representative σ‐SiH complexes (and σ‐BH compounds, see Figure 2), making possible a better distinction between the limits of η^2^ and η^1^ coordination modes.

## Computational details

Geometry optimizations of the complexes were performed without symmetry constraints using the Gaussian09^[16]^ optimizer together with Turbomole 7.1^[17]^ energies and gradients at the BP86^[18]^/def2‐TZVPP^[19]^ level of theory using the D3 dispersion correction suggested by Grimme et al.^[20]^ and the resolution‐of‐identity (RI) approximation.^[21]^ This level is denoted as RI‐BP86‐D3/def2‐TZVPP and was chosen due to its good performance to understand the bonding situation of different transition metal complexes.^[22]^ Vibrational analysis was performed to ensure that the optimized geometry corresponds to an energy minimum. Natural Bond Order (NBO) calculations were performed with the NBO6.0 program^[14]^ at the same BP86‐D3/def2‐TZVPP level.

The interaction ΔE_int_ between the selected fragments is analyzed with the help of the Energy Decomposition Analysis (EDA) method.^[13]^ Within this approach, ΔE_int_ can be decomposed into the following physically meaningful terms [Eq. 1]:

(1) $$\Delta E{}_{\mathrm{int}}=\Delta E{}_{\mathrm{elstat}}+\Delta E{}_{\mathrm{Pauli}}+\Delta E{}_{\mathrm{orb}}+\Delta E{}_{\mathrm{disp}}\;$$

The term ΔE_elstat_ corresponds to the classical electrostatic interaction between the unperturbed charge distributions of the deformed reactants and is usually attractive. The Pauli repulsion ΔE_Pauli_ comprises the destabilizing interactions between occupied orbitals and is responsible for any steric repulsion. The orbital interaction ΔE_orb_ accounts for electron‐pair bonding, charge transfer (interaction between occupied orbitals on one moiety with unoccupied orbitals on the other, including HOMO‐LUMO interactions), and polarization (empty‐occupied orbital mixing on one fragment due to the presence of another fragment). Finally, the ΔE_disp_ term takes into account the interactions which are due to dispersion forces. Moreover, the NOCV (Natural Orbital for Chemical Valence)^[14]^ extension of the EDA method has been also used to further partition the ΔE_orb_ term. The EDA‐NOCV approach provides pairwise energy contributions for each pair of interacting orbitals to the total bond energy.

The program package AMS 2020.10^[23]^ was used for the EDA‐NOCV calculations at the same BP86‐D3 level, in conjunction with a triple‐ζ‐quality basis set using uncontracted Slater‐type orbitals (STOs) augmented by two sets of polarization functions with a frozen‐core approximation for the core electrons.^[24]^ Auxiliary sets of s, p, d, f, and g STOs were used to fit the molecular densities and to represent the Coulomb and exchange potentials accurately in each SCF cycle.^[25]^ Scalar relativistic effects were incorporated by applying the zeroth‐order regular approximation (ZORA).^[26]^ This level of theory is denoted as ZORA‐BP86‐D3/TZ2P//RI‐BP86‐D3/def2‐TZVPP.

## Results and Discussion

We first focused on the parent platinum(II)‐cationic complex **1**, recently prepared by us.^[8b]^ The computed optimized geometry of this species (RI‐BP86‐D3/def2‐TZVPP level) concurs quite well with the experimental structure (X‐ray diffraction), and particularly, the calculated key Pt−Si bond length (2.547 Å) accurately matches the observed value of 2.53(1) Å. According to the NBO method, the corresponding Wiberg Bond Index of this Pt−Si bond is not negligible (WBI=0.274), which confirms a substantial interaction between the silicon atom and the transition metal. Indeed, the Second Order Perturbation Theory (SOPT) of the NBO method indicates that there is a significant stabilizing interaction involving the donation of electron density from a doubly occupied d atomic orbital of the platinum to the vacant σ*(Si−H) molecular orbital (associated SOPT energy, ΔE^(2)^=−16.8 kcal/mol), which supports a substantial backdonation in this species.

**Figure 3:**
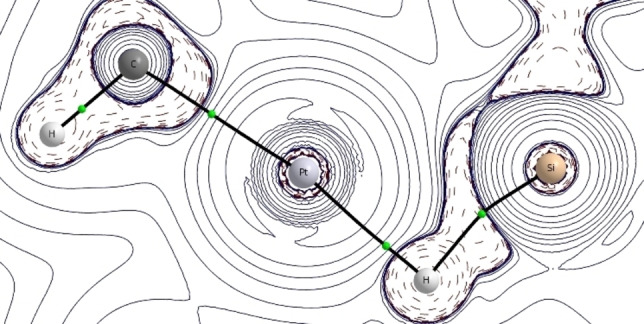
Contour line diagrams ∇^2^ρ(r) for complex **1** in the Pt−H−Si plane. The solid lines connecting the atomic nuclei are the bond paths, while the small green spheres indicate the corresponding bond critical points, respectively.

The NBO data sharply contrast with the previously reported AIM data which did not locate either a BCP or BP running between the platinum and silicon atoms, thus suggesting a low or negligible backdonation. It may be argued that this is the result of known issues in QTAIM about the use of pseudo‐potentials to describe the transition metal.^[27]^ To discard this possibility, we repeated the AIM analysis on **1** using a full‐electron basis‐set to describe platinum (BP86‐D3/6‐31G*&WTBS//RI‐BP86‐D3/def2‐TZVPP level). Our calculations confirm once again the absence of BCP (or BP) between Pt and Si atoms, therefore ruling out issues associated with the description of the third‐row transition metal (Figure 3).

To solve this apparent contradiction between the NBO and QTAIM data, we applied a different yet complementary approach, namely the EDA‐NOCV method. From the data in Table 1, computed at the relativistic ZORA‐BP86‐D3/TZ2P//RI‐BP86‐D3/def2‐TZVPP level, the main contribution to the total interaction (ΔE_int_) between the [Pt]^+^ and SiHEt_3_ fragments in complex **1** comes from the electrostatic attractions (ΔE_elstat_), which represent ca. 58 % of the total attractive interactions, and are almost twice as strong as the orbital interactions (ΔE_orb_). Despite that, the orbital attractions between these fragments are also significant and contribute ca. 32 % to the total bonding. At variance, the stabilizing interactions coming from dispersion forces (ΔE_disp_) are comparatively much weaker (ca. 10 % to the total bonding) but not negligible as observed in relatively bulky transition metal complexes.^[28]^

**Table 1:** Energy Decomposition Analysis (in kcal/mol) and computed WBIs for complexes **1**–**9**.^[a]^

	**1**	**2**	**3**	**4**	**5**	**6 a 6 b**	**7**	**8 a 8 b**	**9 a 9 b**
ΔE_int_	−59.3	−42.6	−97.5	−74.5	−74.4	−33.4 −38.4	−43.4	−43.8 −47.1	−47.9 −51.0
ΔE_Pauli_	203.4	74.3	222.31	141.3	159.2	69.9 72.1	97.9	165.6 157.1	186.7 186.3
ΔE_elstat_	−151.3	−49.1	−162.4	−102.5	−123.5	−49.2 −54.3	−62.3	−120.5 −110.4	−133.3 −135.1
ΔE_orb_	−85.1	−44.9	−138.5	−97.7	−97.1	−44.3 −44.5	−55.1	−71.5 −67.8	−86.6 −83.1
ΔE_orb_(1)^[b]^	−41.8	−28.6	−45.8	−27.6	−41.2	−27.3 −26.9	−38.5	−31.8 −30.1	−29.3 −26.8
ΔE_orb_(2)^[b]^	−20.7	−5.0	−66.8	−59.4	−42.3	−10.5 −10.7	−6.6	−22.0 −17.9	−38.0 −37.0
ΔE_disp_	−26.3	−22.9	−18.9	−15.6	−13.1	−9.8 −11.7	−23.8	−17.4 −25.9	−14.7 −19.2
WBI (M−Si or M−B)	0.27	0.04	0.43	0.53	0.33	0.14 0.15	0.03	0.31/0.27	0.43/0.38
r (Si⋅⋅⋅H or B⋅⋅⋅H) [Å]	1.725	1.585	1.870	1.831	1.803	1.589 1.590	1.613	1.305/1.292	1.329/1.343
WBI (Si−H or B−H)	0.50	0.61	0.28	0.31	0.33	0.55 0.59	0.58	0.69/0.71	0.65/0.64

[a] All data have been computed at the ZORA‐BP86‐D3/TZ2P//RI‐BP86‐D3/def2‐TZVPP level. [b] ΔE_orb_(1) refers to the strength of the donation from the σ(E−H) molecular orbital whereas ΔE_orb_(2) refers to the strenght of the backdonation from the transition metal.

**Figure 4:**
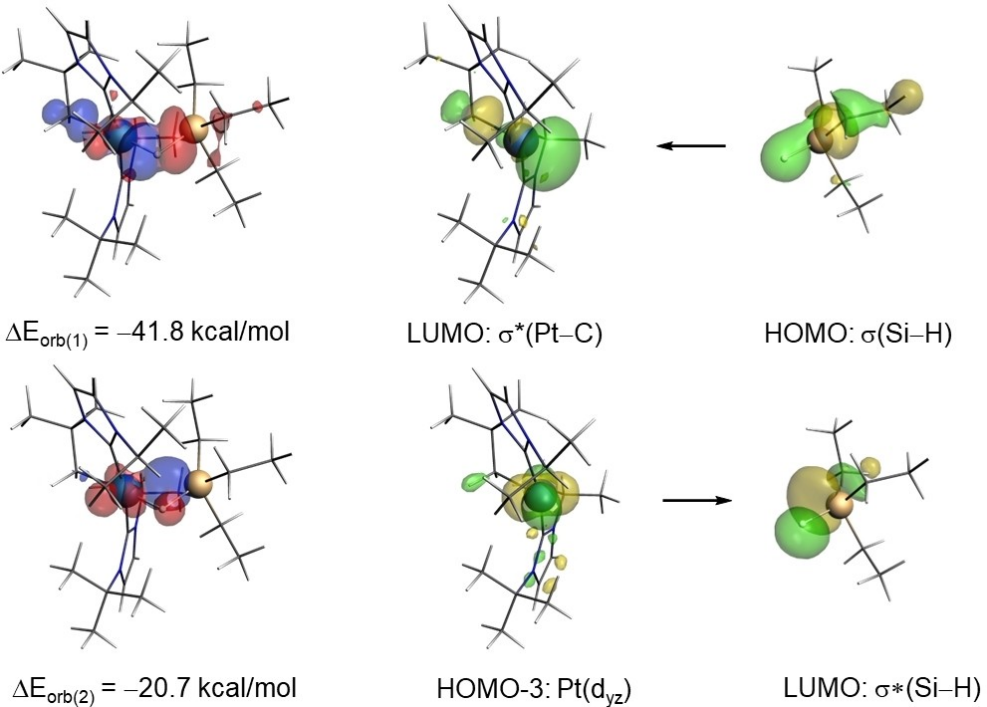
Deformation densities and associated molecular orbitals of the most important orbital interactions, ΔE_orb_(1) and ΔE_orb_(2), in complex **1**. The color code used to represent the flow of charge is red→blue. All data were computed at the ZORA‐BP86‐D3/TZ2P//RI‐BP86‐D3/def2‐TZVPP level.

Further quantitative insight into the nature of the orbital interactions between the [Pt]^+^ and SiHEt_3_ fragments in complex **1** can be gained by means of the NOCV extension of the EDA method. This approach identifies two main orbital interactions which dominate the total ΔE_orb_ term, namely the donation from σ(Si−H) molecular orbital of the SiHEt_3_ ligand to the vacant σ*(Pt−C) molecular orbital of the [Pt]^+^ fragment (denoted ΔE_orb_(1)) and the backdonation from a doubly‐occupied d atomic orbital of the transition metal to the σ*(Si−H) molecular orbital (denoted ΔE_orb_(2)). Hence, the bonding situation in complex **1** can be safely described in terms of the Dewar‐Chatt‐Duncanson (DCD) model^[29]^ with two dative bonds, that is, the σ(Si−H)→σ*(Pt−C) σ‐donation and the d(Pt)→σ*(Si−H) backdonation (Figure 4). Although the computed associated stabilizing energies indicate that the σ‐donation is almost twice as strong as the backdonation (see Table 1), our EDA‐NOCV calculations firmly confirm the occurrence of a significant Pt→Si backdonation in complex **1**, which is in line with the NBO data. Therefore, it can be concluded that one should be particularly cautious when not observing a BCP (and BP) in the QTAIM calculations, which might (inaccurately) suggest that complex **1** is best described as a η^1^‐species.

The crucial role of the backdonation in the bonding of complex **1** is further supported by additional calculations on the analogous system where the Pt−H−Si angle was widened to 140° (vs. 96.5° in **1**). In this situation, the backdonation is dramatically reduced (ΔE_orb_(2)=−5.5 kcal/mol), which is translated into a much weaker interaction between the transition metal fragment and the silane ligand (ΔE_int_=−37.9 kcal/mol). As a result, this species is 3.8 kcal/mol less stable than **1** which highlights the role of backdonation in the stability of the complex.

Once the bonding situation of the parent complex **1** has been clarified, we compare the bonding in the cationic complexes **2** ^[7]^ and **3**,^[30]^ which are typically considered as η^1^ and η^2^ species, respectively. Simple inspection of the M−Si Wiberg Bond Indices derived from the NBO method is in line with this description: whereas **2** presents an almost negligible WBI(Ir−Si) of 0.04, the corresponding WBI(Ru−Si) in **3** is significantly higher (0.43). This directly indicates that, according to the computed WBIs, the parent Pt(II) complex **1** (WBI=0.27) presents a bonding situation that is intermediate between the extreme situations represented by complexes **2** and **3**. Our EDA‐NOCV calculations are fully consistent with this. As shown in Table 1, the total interaction energy (ΔE_int_) between the cationic transition metal fragment and the silane ligand is much stronger (more than twice as strong) in complex **3** than in **2**, while the interaction in **1** is intermediate. This is a direct consequence of a drastic reduction of all the attractive interactions in **2**, as the dominant ΔE_elstat_ term as well as the ΔE_orb_ are markedly weaker. In addition, the computed backdonation is particularly enlightening as it clearly confirms that there is essentially no backdonation in complex **2** (ΔE_orb_(2)=−5.0 kcal/mol), whereas backdonation in complex **3** exhibits a value of −66.8 kcal/mol (in part due to the electron‐withdrawing character of the chlorine atoms, which significantly increase the acceptor ability of the silane ligand). Once again, the corresponding backdonation in the parent complex **1** is intermediate between these two complexes (ΔE_orb_(2)=−20.7 kcal/mol). Not surprisingly, this trend is nicely reflected in the corresponding Si−H bond length in the silane ligand, which increases in the order 1.585 Å (**2**) <1.725 Å (**1**) <1.870 Å (**3**), as a consequence of the population of the σ*(Si−H) molecular orbital. Interestingly, a short Si−H distance of 1.613 Å was computed for the Au(III) cationic complex **7**, described by Bochmann and co‐workers recently.^[31]^ This suggests that the coordination mode in this Au(III)‐species resembles that of **2** and therefore it should be described as a η^1^‐species, which is once again supported by the rather low backdonation computed for this complex (ΔE_orb_(2)=−6.6 kcal/mol).

Once the bonding situation in the above late transition metal complexes has been analyzed, we turned our attention to related complexes involving early transition metals. According to the computed M−Si WBIs, both the formally Ti(II)‐complex **4** ^[32]^ and Mn(I)‐complex **5** ^[11]^ exhibit a high WBI value (0.53 and 0.33, respectively) and long Si−H bond distances (1.831 and 1.803 Å, respectively), comparable to the data observed for complex **3**. This suggests that these species should have a high degree of backdonation and should be viewed as η^2^‐complexes. Indeed, the EDA‐NOCV method confirms that both complexes exhibit high ΔE_orb_(2) values (−59.4 and −42.3 kcal/mol, respectively), thus supporting a remarkable [M]→σ*(Si−H) backdonation. This finding contrasts with the previously reported QTAIM calculations on complex **5** which once again did not locate the expected BCP (or BP) running between the transition metal and silicon atoms.^[11]^ Similarly, a BCP was neither observed in the QTAIM calculations involving the Cr(0)‐complex **6 a**,^[11]^ which could be indicative of η^1^‐coordination mode. Our NBO calculations indicate that the WBI(Cr−Si) is rather low (0.14) and is associated with a short Si−H bond length (1.589 Å), thus strongly suggesting a low degree of backdonation. Once again, the EDA‐NOCV supports this as the computed ΔE_orb_(2) value is comparatively low (−10.5 kcal/mol), which suggests a mainly η^1^‐coordination mode in this complex. Rather similar values were computed for its tungsten counterpart **6 b**, thus indicating a negligible influence of the transition metal on the bonding of these pentacarbonyl complexes.

**Figure 5:**
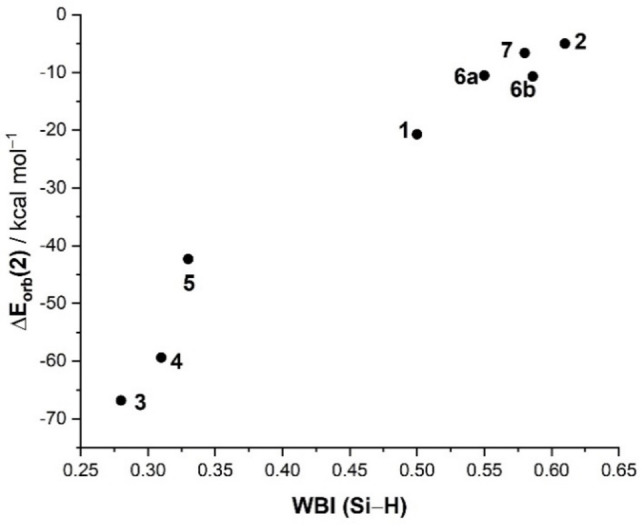
Plot of the computed WBI (Si−H) and the strength of the backdonation ΔE_orb_(2) for complexes **1**–**7**.

The results above show that the extent of the backdonation directly correlates with the Si−H bond length in the sense that complexes exhibiting strong [M]→σ*(Si−H) backdonations (i.e., η^2^‐complexes) are associated with long Si−H distances or lower WBIs, whereas complexes with weak or negligible backdonation present much shorter Si−H bonds (or higher WBIs). For this reason, and despite the fact that the Si−H distance also depends on the depopulation of the σ‐(Si−H) bond, a very good correlation between the computed ΔE_orb_(2) values (which are a direct measure of the strength of the backdonation) and the computed WBI(Si−H) values was found (Figure 5). A similar correlation was found when using the sum of the donation and backdonation energies, which confirms that both orbital interactions influence the Si−H bond (see Figure S1 in the Supporting Information).

**Figure 6:**
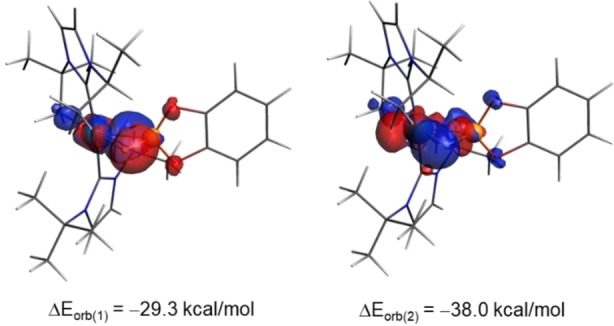
Deformation densities in complex **9 a**. The color code used to represent the flow of charge is red→blue. All data were computed at the ZORA‐BP86‐D3/TZ2P//RI‐BP86‐D3/def2‐TZVPP level.

With all the information described above in hand, we turned our attention to the study of the experimentally described Pt(II) cationic σ‐B−H complexes **8** and **9** ^[33]^ to gain more insight into the bonding situation of these analogous σ‐borane complexes. As in the case of the silane analogue **1**, no BCPs or BPs were observed in the QTAIM analyses^[33]^ and, therefore, these systems are good candidates to explore to potential of EDA‐NOCV methods in elucidating the bonding situation in these σ‐BH complexes. Similar to their Si−H counterpart **1**, our calculations indicate that the electrostatic attractions between the transition metal fragment and the borane ligand constitute the major contributor to the total bonding interactions (ΔE_elstat_ contribution of ca. 57–59 %, Table 1). Despite that, the orbital interactions (measured by the ΔE_orb_ term) are also significant and, according to the NOCV approach, mainly consist of the donation from the σ(B−H) molecular orbital of the borane ligand to the vacant σ*(Pt−C) molecular orbital of the [Pt]^+^ fragment and the backdonation from a doubly‐occupied d atomic orbital of the transition metal to the empty p_z_ orbital on boron (Figure 6). This is different from that observed for silane complexes (where backbonding takes place at the σ*(Si−H) orbital), yet it is in good agreement with previous DFT calculations on **8 a**;^[33b]^ in this complex, closing the Pt−H−B angle to ca. 68° leads to borane dissociation instead of B−H oxidative addition, in line with the lack of participation of the σ*(B−H) orbital, which suggests a σ‐complex assisted metathesis (σ‐CAM) type mechanism for the activation of the B−H bond.^[34]^ From the data in Table 1, it can be concluded that the σ‐donation coming from B−H bonds is comparatively weaker than that coming from Si−H bonds whereas the d(Pt)→p_z_(B) backdonation seems, in general, stronger. Therefore, it can be concluded that borane ligands are, in this type of Pt(II)‐complexes, better acceptors but poorer donors than silane ligands. Moreover, according to the computed backdonation strengths (ca. −20 to −30 kcal/mol) and the relatively high WBIs (Pt−B) (0.3–0.4), these compounds can be considered as having an intermediate bonding situation between the extreme η^1^ and η^2^‐coordination.

For completeness, we finally explore the bonding situation in analogous σ‐(Si−H) complexes involving main‐group systems (see Figure 2), where, in principle, the backdonation should be minimal and therefore should be considered as genuine η^1^‐species. From the data in Table 2, it becomes evident that, at variance with their transition metal counterparts, the bonding situation in compounds **10**–**13** is dominated by the orbital interactions, which are nearly twice as strong as the electrostatic interactions. In addition, it is confirmed that the main orbital interaction in these species derives almost exclusively from the σ‐donation coming from the silane to the vacant p_z_ atomic orbital of the main group element (ΔE_orb_(1)). Expectedly, the backdonation into the σ*(Si−H) molecular orbital (ΔE_orb_(2)) can be considered as practically negligible (ca. −5 kcal/mol), which supports the η^1^‐coordination mode in these silane‐complexes.

**Table 2:** Energy Decomposition Analysis (in kcal/mol) and Si−H bond lengths for complexes **10**–**13**.^[a]^

	**10**	**11 a**	**11 b**	**12**	**13**
ΔE_int_	−52.1	−26.5	−28.8	−35.6	−58.7
ΔE_Pauli_	65.9	85.3	46.5	99.5	124.4
ΔE_elstat_	−38.2	−36.3	−25.2	−42.4	−59.8
ΔE_orb_	−73.5	−56.3	−33.0	−70.3	−106.2
ΔE_orb_(1)	−63.0	−44.2	−25.2	−55.3	−85.8
ΔE_orb_(2)	−4.4	−6.6	−3.0	−7.6	−6.6
ΔE_disp_	−6.4	−19.2	−17.1	−22.4	−17.0
r (Si⋅⋅⋅H) [Å]	1.634	1.582	1.536	1.592	1.655

^[a]^ All data have been computed at the ZORA‐BP86‐D3/TZ2P//RI‐BP86‐D3/def2‐TZVPP level.

A closer inspection of the data in Table 2 reveals interesting trends in the bonding situation of compounds **10**–**13**. On one hand, the higher interaction between the silane and the main‐group fragment in **11 b** as compared to **11 a** indicates that aluminum is a better acceptor than boron, which agrees with the well‐known higher Lewis acidity of Al(C_6_F_5_)_3_ with respect to B(C_6_F_5_)_3_.^[35]^ Despite that, the acceptor ability of the boron fragment is significantly enhanced in the borole **12**, which exhibits a markedly stronger interaction (ΔE_int_=−35.6 kcal/mol vs. −26.5 kcal/mol, for **12** and **11 a**, respectively). This is in part the result of the reduction of the antiaromaticity of the borole fragment upon binding with the silane ligand. Indeed, the Nuclear Independent Chemical Shift (NICS)^[36]^ value computed in the borole fragment becomes less positive (i.e., less antiaromatic) when going from the naked (i.e., non‐bonded) borole (NICS=+13.5 ppm) to the silane‐bonded borole **12** (NICS=+4.7 ppm). A similar finding was found by some of us on the antiaromaticity‐enhanced reactivity of related frustrated Lewis pairs having borole fragments as the Lewis acid partner.^[37]^ Finally, cationic systems **10** and **13** exhibit the strongest interactions of the entire series. Certainly, this is due to the enhanced acceptor nature of the main‐group fragment which is reflected in the computed high values of the donation from the σ‐(Si−H) of the silane (ΔE_orb_(1)). Species **13** deserves some additional comments. This compound has been described as a η^2^‐SiH species, mainly because of the relatively short B⋅⋅⋅Si distance of 2.570(6) Å and a relatively acute B−H−Si angle (126(4)°) (compared to the boron derivative **11 a**, 157°).^[4a]^ According to the authors, neither BCPs nor bond paths are present in the AIM analysis whereas NBO calculations suggests negligible back‐donation into the σ*(Si−H) bond. Our results are in line with these observations and describe better compound **13** as an η^1^ rather than an η^2^ species.

**Figure 7:**
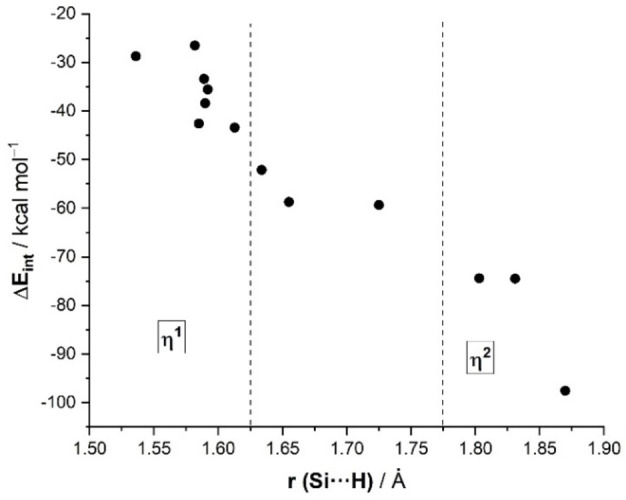
Plot of the computed Si⋅⋅⋅H bond length and the total interaction (ΔE_int_) in complexes **1**–**13**.

By combining the data in Tables 1 and 2, it becomes evident that complexes having the η^2^‐coordination mode exhibit much stronger interaction energies between the transition metal/main‐group fragment and the silane ligand than compounds featuring η^1^‐coordination or intermediate situations. In addition, η^2^‐complexes are also associated with long Si⋅⋅⋅H bond lengths as a consequence of both the depopulation of the σ(Si−H) molecular orbital and the population, by backdonation, of the corresponding σ*(Si−H) molecular orbital. For this reason, it is not surprising that a good correlation was found when plotting both parameters (Figure 7). From the data in Figure 7, there appears to exist a limit defining the coordination mode: systems having ΔE_int_≥|−70| kcal/mol can be safely characterized as η^2^‐systems, whereas much lower values (ΔE_int_≤|−40| kcal/mol) are expected for η^1^‐complexes. Systems having values between these values present intermediate bonding situations, such as the Pt(II)‐complex **1**, or belong to η^1^‐species where the fragment exhibits remarkably high acceptor abilities, such as the cationic species **10** or **13**.

## Conclusion

In summary, the combination of EDA‐NOCV calculations with AIM and NBO methods constitutes a powerful tool for analyzing the interaction of E−H bonds with both transition metals and *p*‐block Lewis acids. This is particularly helpful in those cases where QTAIM analysis is not able to locate either bond critical points or bond paths between the key atoms involved in the bonding. At variance, our EDA‐NOCV calculations make it possible to not only identify but also quantify the two possible components of the σ‐SiH (and σ‐BH) bond interaction (i.e. donation and backdonation) allowing us to establish a scale that represents a continuum between genuine η^1^ and η^2^ interactions. Therefore, this contribution sheds light on the intrinsic ambiguity of the designation of complexes as η^1^ and η^2^ in the sense that not all these types of interactions can be labelled as belonging to these extreme situations but a whole of different intermediate possibilities are in between.

## Conflict of interest

The authors declare no conflict of interest.

1

## Supporting information

As a service to our authors and readers, this journal provides supporting information supplied by the authors. Such materials are peer reviewed and may be re‐organized for online delivery, but are not copy‐edited or typeset. Technical support issues arising from supporting information (other than missing files) should be addressed to the authors.

Supporting Information

## Data Availability

Research data are not shared.
