# Supplementary material for: Bonding Situation of σ‐E−H Complexes in Transition Metal and Main Group Compounds
Source: Chemistry. 2022 Aug 22;28(57):e202201920. doi: 10.1002/chem.202201920 (PMC9804526; doi:10.1002/chem.202201920)
Supplement: Supplementary file 1 — Supporting Information [file CHEM-28-0-s001.pdf]

Chemistry–A European Journal

Supporting Information

Bonding Situation of σ-E–H Complexes in Transition Metal and Main Group Compounds

Pablo Ríos, Salvador Conejero,* and Israel Fernández*

Contents

1. Figure S1……………………………………………………………………………1
2. Coordinates of the optimized structures………………………………………………2
3. References…………………………………………………………………………...29

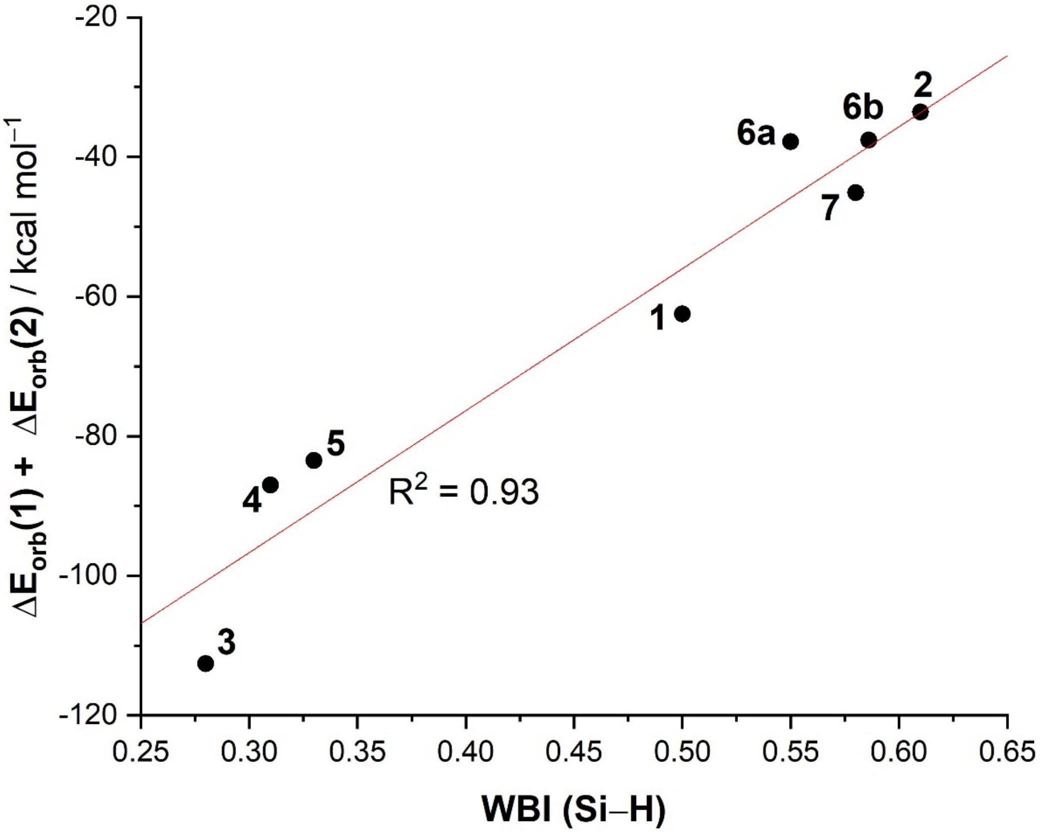

Figure S1. Correlation between the computed WBI (Si-H) and the sum of the donation ($\Delta E_{orb}(1)$) and backdonation ($\Delta E_{orb}(2)$) energies for complexes **1-7**.

2. Cartesian coordinates of the optimized structures

Complex 1[1]

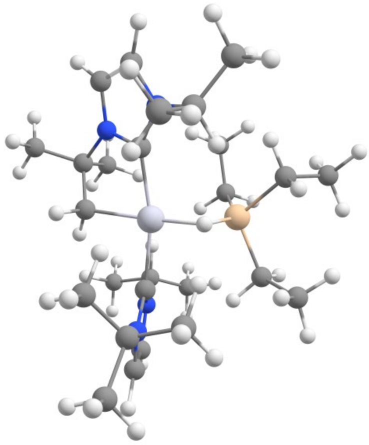

C	-1.999143000	-0.669196000	0.427053000
C	-4.209204000	-0.916535000	0.801016000
H	-5.210842000	-0.623567000	1.084120000
C	-3.728358000	-2.124808000	0.383058000
H	-4.237185000	-3.066831000	0.230183000
C	-1.322197000	-2.945285000	-0.178069000
C	-1.664735000	-4.304152000	0.441768000
H	-1.833324000	-4.213954000	1.522574000
H	-0.823933000	-4.990324000	0.281666000
H	-2.552445000	-4.759195000	-0.019399000
C	-0.027859000	-2.354664000	0.424554000
H	0.844165000	-2.832511000	-0.037199000
H	0.011513000	-2.582610000	1.501618000
C	-3.150226000	1.304824000	1.451314000
H	-2.283980000	1.820185000	1.022928000
C	-4.421210000	2.071238000	1.097643000
H	-4.597843000	2.080326000	0.014571000
H	-4.325111000	3.109116000	1.438298000
H	-5.305193000	1.646193000	1.592059000
C	-2.932965000	1.164068000	2.959853000
H	-3.755548000	0.603319000	3.424253000
H	-2.886989000	2.155621000	3.427804000
H	-1.991010000	0.637971000	3.162895000
C	2.039980000	-0.271044000	0.233296000
C	4.166598000	-0.627764000	-0.468504000
H	4.955333000	-0.927099000	-1.145262000
C	4.230790000	-0.120099000	0.789633000
H	5.096135000	0.096741000	1.396761000
C	2.568323000	0.585928000	2.599722000
C	1.531477000	-0.355552000	3.227713000
H	0.582917000	-0.327024000	2.670539000
H	1.332370000	-0.041278000	4.259782000
H	1.895125000	-1.390967000	3.238859000
C	2.022467000	2.013298000	2.485665000

H	2.759293000	2.673878000	2.011708000
H	1.799014000	2.405026000	3.486043000
H	1.099062000	2.033173000	1.896725000
C	3.825947000	0.592607000	3.480454000
H	4.274495000	-0.405942000	3.563949000
H	3.535900000	0.912285000	4.488137000
H	4.582753000	1.302419000	3.122404000
C	2.330601000	-1.318795000	-2.051228000
H	1.238645000	-1.295216000	-1.948125000
C	2.788012000	-2.773845000	-2.171034000
H	3.875387000	-2.844206000	-2.309845000
H	2.310973000	-3.237539000	-3.043500000
H	2.518312000	-3.352878000	-1.279320000
C	2.750669000	-0.480821000	-3.257936000
H	2.402205000	0.553553000	-3.169782000
H	2.322672000	-0.911320000	-4.171858000
H	3.842941000	-0.469642000	-3.374057000
C	-1.538707000	3.074236000	-1.139749000
H	-2.510168000	2.591104000	-0.962423000
H	-1.330579000	3.684842000	-0.247410000
C	-1.637285000	3.971607000	-2.388734000
H	-0.681246000	4.460048000	-2.621742000
H	-2.383568000	4.765697000	-2.250263000
H	-1.933698000	3.398542000	-3.277979000
C	1.468183000	2.588748000	-1.545441000
H	2.269082000	1.878173000	-1.298947000
H	1.583359000	2.814751000	-2.618298000
C	1.639049000	3.877673000	-0.722937000
H	0.957953000	4.668792000	-1.061751000
H	2.661769000	4.270467000	-0.804538000
H	1.435337000	3.713027000	0.342798000
C	-0.707422000	0.733038000	-2.857012000
H	-0.451823000	1.400365000	-3.699242000
H	-0.063820000	-0.146788000	-2.978241000
C	-2.188473000	0.336849000	-2.936785000
H	-2.489941000	-0.286258000	-2.085113000
H	-2.398901000	-0.231249000	-3.853204000
H	-2.843436000	1.218016000	-2.943418000
C	-1.228623000	-3.056190000	-1.705579000
H	-2.180950000	-3.388751000	-2.138877000
H	-0.453999000	-3.784533000	-1.977565000
H	-0.968256000	-2.086926000	-2.146087000
N	2.925471000	0.084571000	1.221230000
N	2.824552000	-0.725236000	-0.789672000
N	-3.136452000	-0.035007000	0.825387000
N	-2.374223000	-1.955133000	0.176489000
Si	-0.207613000	1.737102000	-1.318214000
Pt	-0.025032000	-0.256371000	0.257028000
H	-0.143369000	1.423921000	0.377159000

Complex 2[2]

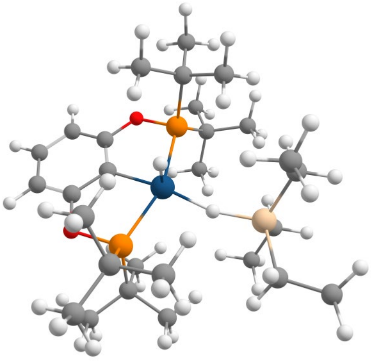

Ir	-0.057497000	-0.193978000	0.047030000
H	-0.088770000	-0.098724000	1.585750000
P	-2.395578000	-0.012599000	-0.015702000
P	2.036150000	-1.192293000	0.134255000
O	-2.889753000	-1.564941000	-0.402963000
C	-1.907964000	-2.534935000	-0.345796000
C	-0.575858000	-2.138729000	-0.117297000
C	0.386886000	-3.167203000	-0.056874000
O	1.710452000	-2.829129000	0.162703000
C	-2.273601000	-3.868483000	-0.517083000
H	-3.316516000	-4.124675000	-0.693142000
C	-1.278602000	-4.848242000	-0.451887000
H	-1.550268000	-5.894971000	-0.581979000
C	0.058900000	-4.510627000	-0.223646000
H	0.839092000	-5.267830000	-0.176554000
C	-3.164430000	0.898589000	-1.468225000
C	-2.155910000	0.679822000	-2.618500000
H	-2.039142000	-0.388005000	-2.850013000
H	-1.165220000	1.095241000	-2.383929000
H	-2.520702000	1.181608000	-3.526842000
C	-3.288758000	2.391250000	-1.129508000
H	-3.512273000	2.956489000	-2.045987000
H	-2.363614000	2.801122000	-0.705004000
H	-4.103142000	2.580114000	-0.419156000
C	-4.527647000	0.333329000	-1.904512000
H	-5.312720000	0.499011000	-1.160789000
H	-4.472524000	-0.739598000	-2.118701000
H	-4.833990000	0.849074000	-2.826934000
C	-3.302007000	0.209917000	1.618400000
C	-2.889713000	1.535627000	2.271489000
H	-3.220394000	2.409386000	1.697117000
H	-1.802870000	1.591926000	2.405325000
H	-3.352861000	1.603531000	3.266464000
C	-4.827726000	0.141316000	1.446385000
H	-5.139164000	-0.768038000	0.917888000
H	-5.223969000	1.015687000	0.916023000
H	-5.293761000	0.126266000	2.442553000
C	-2.843685000	-0.959184000	2.517591000

H	-3.321575000	-0.845350000	3.501149000
H	-1.756923000	-0.965971000	2.671087000
H	-3.137541000	-1.933741000	2.110192000
C	2.998160000	-1.088141000	-1.479809000
C	1.905254000	-1.197008000	-2.568359000
H	1.165552000	-0.382142000	-2.493911000
H	1.366968000	-2.152336000	-2.518368000
H	2.373885000	-1.120899000	-3.560490000
C	4.000429000	-2.237606000	-1.673402000
H	4.374876000	-2.200951000	-2.707297000
H	3.531354000	-3.216116000	-1.519152000
H	4.866619000	-2.157034000	-1.009733000
C	3.698236000	0.272545000	-1.581923000
H	4.518435000	0.371845000	-0.859709000
H	2.992982000	1.098481000	-1.429967000
H	4.127282000	0.385318000	-2.588175000
C	3.052633000	-1.021703000	1.696504000
C	2.200854000	-1.642312000	2.824035000
H	2.048311000	-2.717521000	2.677049000
H	1.216249000	-1.162671000	2.908697000
H	2.727157000	-1.495768000	3.778045000
C	4.400643000	-1.754706000	1.611194000
H	4.876101000	-1.735149000	2.602718000
H	5.088995000	-1.268257000	0.909462000
H	4.279099000	-2.805242000	1.319541000
C	3.266095000	0.472618000	1.984554000
H	2.308512000	0.998595000	2.091394000
H	3.850313000	0.971644000	1.202556000
H	3.817452000	0.579266000	2.929748000
H	0.657949000	1.472036000	0.100267000
Si	0.756469000	3.043170000	-0.085615000
C	0.281498000	3.486103000	-1.847350000
H	-0.778351000	3.261867000	-2.022801000
H	0.357874000	4.586040000	-1.895680000
C	1.158310000	2.849015000	-2.936539000
H	2.219355000	3.101997000	-2.806025000
H	0.860635000	3.188651000	-3.937290000
H	1.081381000	1.752919000	-2.926601000
C	-0.317973000	3.808311000	1.245610000
H	-0.297049000	4.899574000	1.089371000
H	-1.361833000	3.507014000	1.090053000
C	0.145968000	3.464258000	2.671462000
H	0.235733000	2.377183000	2.817869000
H	-0.558978000	3.840142000	3.424483000
H	1.127651000	3.903984000	2.891986000
C	2.565423000	3.452643000	0.239886000
H	2.814217000	3.172166000	1.272123000
H	3.224146000	2.858956000	-0.407119000
C	2.824806000	4.958414000	0.022076000
H	2.650687000	5.257237000	-1.020620000

H	3.865045000	5.212914000	0.264918000
H	2.181459000	5.582301000	0.658338000

Complex 3[3]

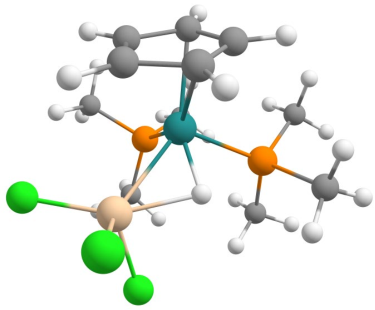

Ru	-0.402095000	35.732642000	-1.648941000
P	-2.276989000	35.587813000	-0.275875000
C	-2.231258000	36.573533000	1.267814000
H	-1.425757000	36.237447000	1.926909000
H	-3.191099000	36.474192000	1.792378000
H	-2.067379000	37.630154000	1.027760000
C	-3.757750000	36.251895000	-1.122979000
H	-3.999551000	35.619298000	-1.985995000
H	-3.559718000	37.267698000	-1.484074000
H	-4.612957000	36.271659000	-0.434956000
C	-2.847288000	33.938095000	0.258066000
H	-3.834315000	34.024549000	0.730767000
H	-2.150645000	33.494743000	0.977717000
H	-2.916465000	33.283514000	-0.619449000
P	0.760918000	34.189396000	-0.313274000
C	0.695946000	34.534997000	1.491366000
H	-0.291485000	34.302785000	1.904647000
H	0.922420000	35.591418000	1.677018000
H	1.439566000	33.913613000	2.007627000
C	0.420158000	32.392479000	-0.360759000
H	0.755763000	31.979033000	-1.318120000
H	-0.647879000	32.183570000	-0.253952000
H	0.975120000	31.909820000	0.454478000
C	2.574922000	34.224455000	-0.580123000
H	2.803333000	34.096151000	-1.642702000
H	3.037153000	33.408815000	-0.009044000
H	2.986099000	35.176027000	-0.224338000
C	0.684695000	37.591911000	-0.972736000
H	1.009362000	37.799973000	0.041358000
C	0.694350000	36.975649000	-3.210104000
H	1.016929000	36.597400000	-4.174607000
C	-0.562155000	37.606084000	-2.932150000
H	-1.362327000	37.761633000	-3.648454000
C	-0.558763000	37.997401000	-1.562423000
H	-1.355268000	38.539890000	-1.061762000
C	1.460468000	36.967342000	-2.009450000
H	2.472752000	36.592838000	-1.918716000
Si	-0.478443000	34.068306000	-3.359371000

H	-1.693160000	35.057210000	-2.338244000
Cl	1.404705000	33.322438000	-3.868302000
Cl	-1.657886000	32.384227000	-3.005238000
Cl	-1.225450000	34.865922000	-5.129006000

Complex 4[4]

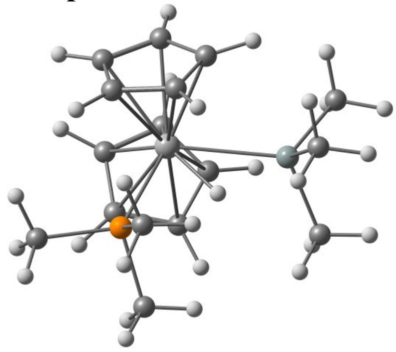

Ti	0.042059000	-0.611017000	0.000058000
Si	-2.089049000	0.963302000	-0.000068000
P	2.049135000	0.906074000	-0.000261000
C	0.001912000	-0.243396000	2.338152000
H	0.004260000	0.761691000	2.742309000
C	1.141266000	-1.061425000	2.097838000
H	2.173727000	-0.806874000	2.312490000
C	0.696527000	-2.297503000	1.577156000
H	1.329988000	-3.126288000	1.279918000
C	-0.723672000	-2.257531000	1.507736000
H	-1.367591000	-3.063382000	1.174966000
C	-1.162733000	-0.987856000	1.974685000
H	-2.189811000	-0.705648000	2.168216000
C	0.695290000	-2.298710000	-1.576338000
H	1.328107000	-3.127817000	-1.278621000
C	1.141014000	-1.063086000	-2.097248000
H	2.173696000	-0.809313000	-2.311785000
C	0.002218000	-0.244374000	-2.338117000
H	0.005249000	0.760544000	-2.742685000
C	-1.162971000	-0.987862000	-1.974450000
H	-2.189832000	-0.705013000	-2.168184000
C	-0.724874000	-2.257670000	-1.507003000
H	-1.369432000	-3.062870000	-1.173889000
C	-2.220317000	2.143817000	-1.498119000
H	-3.115615000	2.774733000	-1.392966000
H	-2.302515000	1.619277000	-2.460184000
H	-1.346287000	2.809170000	-1.555007000
C	3.792747000	0.264190000	0.000133000
H	3.952896000	-0.362481000	-0.886026000
H	3.952858000	-0.361682000	0.886865000
H	4.531397000	1.077913000	-0.000211000
C	2.148871000	2.094962000	1.405993000
H	2.988448000	2.789709000	1.269357000
H	2.282599000	1.560380000	2.353215000
H	1.208907000	2.657362000	1.454634000

C	2.149234000	2.093942000	-1.407344000
H	2.988625000	2.788934000	-1.270901000
H	1.209183000	2.656136000	-1.456813000
H	2.283443000	1.558632000	-2.354097000
H	-0.263578000	1.108756000	-0.000221000
C	-3.794610000	0.071812000	-0.000074000
H	-4.601700000	0.821523000	-0.000447000
H	-3.936637000	-0.565423000	-0.884137000
H	-3.936884000	-0.564783000	0.884413000
C	-2.220533000	2.143809000	1.497961000
H	-1.346202000	2.808706000	1.555501000
H	-3.115416000	2.775218000	1.392206000
H	-2.303738000	1.619233000	2.459918000

Complex 5[5]

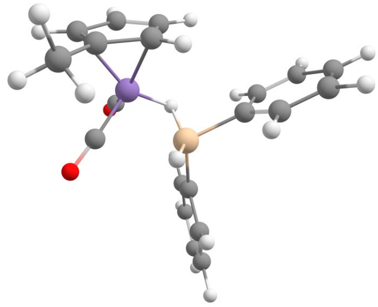

Mn	4.364222000	1.023865000	5.504699000
O	4.933483000	1.555680000	8.344177000
O	1.684466000	2.165014000	5.894395000
C	4.703366000	1.352322000	7.222343000
C	2.764112000	1.752487000	5.740892000
C	3.903208000	-1.064665000	5.493257000
C	5.316175000	-0.896689000	5.648180000
C	5.810873000	-0.218814000	4.500827000
C	4.703229000	0.034095000	3.630355000
C	3.518280000	-0.496206000	4.234521000
C	2.149630000	-0.500287000	3.628432000
H	3.231072000	-1.534958000	6.203557000
H	5.903452000	-1.209248000	6.505582000
H	6.834783000	0.093320000	4.324948000
H	4.759716000	0.512825000	2.657593000
H	1.991377000	0.396177000	3.016361000
H	1.373111000	-0.527396000	4.401951000
H	2.022152000	-1.381836000	2.983220000
Si	4.453956000	2.999356000	4.157239000
C	4.275858000	4.620968000	5.096814000
C	4.892150000	4.834714000	6.342252000
C	4.779530000	6.060691000	7.002184000
C	4.039461000	7.097759000	6.427637000
C	3.413764000	6.902599000	5.193030000
C	3.532872000	5.675636000	4.536164000
C	5.961282000	3.063032000	3.011135000

C	5.795635000	3.149856000	1.618078000
C	6.898514000	3.195105000	0.758939000
C	8.193065000	3.156514000	1.282169000
C	8.379200000	3.072741000	2.666112000
C	7.273236000	3.023576000	3.517094000
H	5.425248000	2.163895000	5.426127000
H	3.294792000	2.948079000	3.198149000
H	5.459810000	4.030405000	6.813643000
H	5.262469000	6.204166000	7.969466000
H	3.944407000	8.053493000	6.944091000
H	2.828628000	7.706215000	4.744192000
H	3.034581000	5.533878000	3.574820000
H	4.788249000	3.183664000	1.196977000
H	6.746689000	3.260614000	-0.319225000
H	9.055070000	3.191464000	0.615301000
H	9.388011000	3.045846000	3.080345000
H	7.435819000	2.952832000	4.595570000

Complex 6a[5]

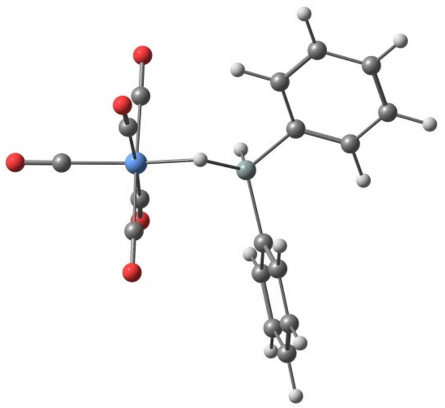

Cr	1.834972000	-0.380002000	0.071136000
C	1.718785000	0.972355000	1.404785000
C	1.807909000	-1.735936000	1.402400000
C	3.693047000	-0.376684000	0.189469000
C	1.893430000	-1.704437000	-1.285937000
C	1.962167000	0.973375000	-1.245741000
O	1.665317000	1.798298000	2.208728000
O	1.814219000	-2.561201000	2.209709000
O	1.950251000	-2.507767000	-2.114953000
O	2.092117000	1.798418000	-2.045892000
O	4.847867000	-0.382860000	0.273724000
H	0.169737000	-0.597634000	0.345347000
Si	-0.808270000	-0.004271000	-0.757715000
H	-0.461920000	-0.079067000	-2.202450000
C	-1.277028000	1.734018000	-0.279696000
C	-1.818655000	1.994292000	0.993197000
C	-1.071502000	2.812357000	-1.158430000
C	-2.141476000	3.295928000	1.378253000
H	-1.990868000	1.171297000	1.689821000
C	-1.396889000	4.115677000	-0.775052000

H	-0.645578000	2.635143000	-2.147165000
C	-1.930256000	4.358163000	0.493453000
H	-2.556329000	3.483518000	2.368817000
H	-1.231020000	4.942581000	-1.465886000
H	-2.180709000	5.375734000	0.794263000
C	-2.147923000	-1.248903000	-0.329893000
C	-3.504404000	-0.879696000	-0.377800000
C	-1.821624000	-2.573185000	0.010858000
C	-4.506373000	-1.813422000	-0.102509000
H	-3.780510000	0.147579000	-0.623137000
C	-2.822148000	-3.508351000	0.284271000
H	-0.774925000	-2.879668000	0.069550000
C	-4.166098000	-3.128530000	0.226765000
H	-5.553889000	-1.513206000	-0.141526000
H	-2.553179000	-4.531425000	0.548010000
H	-4.948295000	-3.856240000	0.444223000

Complex 6b

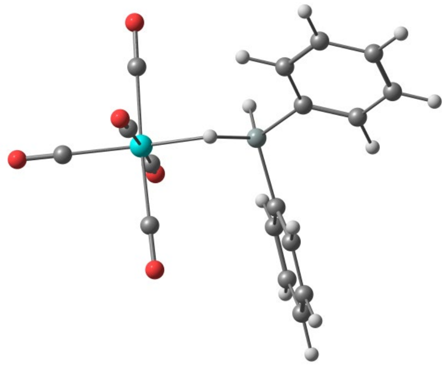

W	1.512374000	-0.263778000	0.047987000
C	1.224104000	1.250185000	1.421641000
C	1.548024000	-1.673195000	1.561077000
C	3.511424000	-0.096255000	0.214226000
C	1.702346000	-1.752175000	-1.362654000
C	1.559494000	1.142383000	-1.448738000
O	1.058425000	2.105526000	2.180042000
O	1.589416000	-2.459910000	2.405708000
O	1.803194000	-2.581884000	-2.162454000
O	1.607252000	1.933277000	-2.292192000
O	4.662425000	-0.013691000	0.326011000
H	-0.300649000	-0.666246000	0.245322000
Si	-1.305858000	-0.014249000	-0.799999000
H	-0.944402000	-0.094830000	-2.240533000
C	-1.747429000	1.724073000	-0.303582000
C	-2.279058000	1.978204000	0.974691000
C	-1.515374000	2.809736000	-1.166881000
C	-2.563826000	3.282033000	1.381227000
H	-2.468960000	1.149244000	1.659365000
C	-1.803932000	4.114846000	-0.761879000
H	-1.096614000	2.637006000	-2.159263000
C	-2.325585000	4.351821000	0.512704000

H	-2.968795000	3.465524000	2.376557000
H	-1.617488000	4.947659000	-1.440231000
H	-2.545440000	5.371137000	0.830889000
C	-2.640318000	-1.254664000	-0.354558000
C	-3.997700000	-0.887613000	-0.382501000
C	-2.305718000	-2.576054000	-0.010102000
C	-4.993304000	-1.820533000	-0.082455000
H	-4.279421000	0.137629000	-0.630015000
C	-3.299862000	-3.510554000	0.287226000
H	-1.256812000	-2.879016000	0.033602000
C	-4.645207000	-3.132668000	0.250477000
H	-6.041796000	-1.522162000	-0.104980000
H	-3.025087000	-4.531256000	0.554208000
H	-5.422365000	-3.859621000	0.487632000

Complex 7[6]

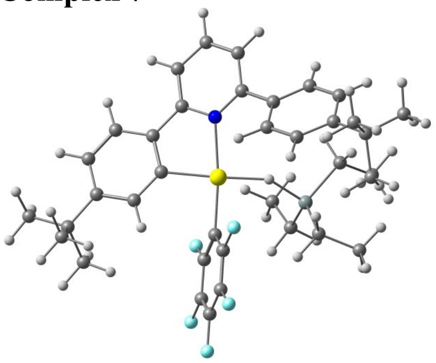

C	-0.911925000	-3.384959000	0.301136000
C	-0.729542000	-4.738165000	0.596400000
C	0.559948000	-5.258223000	0.653298000
C	1.644508000	-4.439200000	0.355065000
C	1.441100000	-3.080619000	0.070935000
N	0.179349000	-2.569870000	0.129105000
H	0.715879000	-6.312209000	0.880734000
H	-1.593322000	-5.377045000	0.762441000
H	2.649881000	-4.847954000	0.286665000
C	2.556773000	-2.228355000	-0.372573000
C	3.842846000	-2.384346000	0.165195000
C	2.384403000	-1.283160000	-1.403526000
C	4.906604000	-1.603202000	-0.284430000
H	4.012419000	-3.097349000	0.972365000
C	3.446456000	-0.497365000	-1.832791000
H	1.420195000	-1.190605000	-1.905649000
C	4.735530000	-0.630365000	-1.281905000
H	5.882460000	-1.750765000	0.172887000
H	3.268702000	0.219984000	-2.633315000
C	-2.202510000	-2.740575000	0.076405000
C	-2.210842000	-1.368948000	-0.260682000
C	-3.435731000	-3.410040000	0.070084000
C	-3.367655000	-0.714504000	-0.634977000
C	-4.610484000	-2.743704000	-0.273663000
H	-3.484547000	-4.468990000	0.323879000

C	-4.606166000	-1.391120000	-0.644931000
H	-3.334252000	0.336076000	-0.909738000
H	-5.543840000	-3.301686000	-0.257849000
C	-5.866691000	-0.615268000	-1.037755000
C	-7.132493000	-1.484615000	-0.956500000
H	-7.308193000	-1.858509000	0.061676000
H	-8.006096000	-0.884250000	-1.241326000
H	-7.085501000	-2.341870000	-1.642237000
C	-5.709500000	-0.104109000	-2.488756000
H	-6.606413000	0.456353000	-2.785397000
H	-4.846837000	0.566789000	-2.597305000
H	-5.580450000	-0.940339000	-3.189021000
C	-6.032864000	0.588202000	-0.079387000
H	-6.934939000	1.154808000	-0.346726000
H	-6.133330000	0.252520000	0.961297000
H	-5.180408000	1.278993000	-0.130624000
Au	-0.389631000	-0.520840000	0.088896000
C	-1.108873000	1.373164000	-0.065589000
C	-2.058252000	1.902273000	0.806830000
C	-0.667932000	2.182231000	-1.110124000
C	-2.557744000	3.198232000	0.653775000
C	-1.142694000	3.483338000	-1.291929000
C	-2.094261000	3.992238000	-0.401413000
F	0.258622000	1.715467000	-1.981416000
F	-2.524614000	1.162078000	1.835132000
F	-3.466515000	3.686689000	1.507624000
F	-0.693637000	4.243093000	-2.299254000
F	-2.560521000	5.233761000	-0.558531000
H	1.198824000	0.115044000	0.628109000
Si	1.877969000	1.164977000	1.646309000
C	3.326129000	0.173038000	2.313249000
H	3.007676000	-0.868758000	2.464131000
H	4.099014000	0.137703000	1.532460000
C	3.889637000	0.756184000	3.625350000
H	4.756842000	0.173991000	3.965178000
H	4.220795000	1.796699000	3.507841000
H	3.145704000	0.736874000	4.432760000
C	2.349296000	2.578309000	0.511489000
H	2.743791000	2.142427000	-0.418692000
H	1.435876000	3.125140000	0.235708000
C	3.381353000	3.535507000	1.140242000
H	3.002239000	4.002473000	2.059705000
H	4.316650000	3.017819000	1.392782000
H	3.635436000	4.343943000	0.442072000
C	0.636021000	1.568481000	2.987498000
H	1.173244000	2.212063000	3.705242000
H	-0.164458000	2.194452000	2.570745000
C	0.056951000	0.339325000	3.707581000
H	0.846603000	-0.298100000	4.129207000
H	-0.605076000	0.636449000	4.530966000

H	-0.540778000	-0.279777000	3.022843000
C	5.876914000	0.260701000	-1.781664000
C	6.085791000	0.013692000	-3.293802000
H	5.187363000	0.253539000	-3.877170000
H	6.904001000	0.644882000	-3.666440000
H	6.347058000	-1.035311000	-3.487351000
C	7.200925000	-0.019359000	-1.051838000
H	7.120645000	0.162749000	0.029309000
H	7.545408000	-1.050969000	-1.207497000
H	7.981102000	0.648132000	-1.439792000
C	5.495558000	1.740613000	-1.548512000
H	4.580214000	2.020504000	-2.086817000
H	5.338856000	1.940715000	-0.479651000
H	6.301898000	2.397370000	-1.901924000

Complex 8a[7]

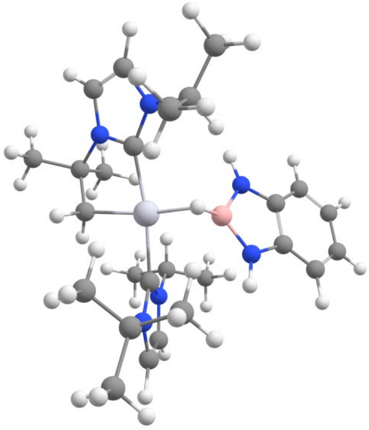

Pt	0.623546000	-0.364500000	-0.094173000
N	3.123735000	0.528719000	1.058401000
N	2.837804000	1.834773000	-0.645842000
N	-1.768377000	-1.784573000	1.119857000
C	2.263907000	0.808059000	0.041628000
C	1.785305000	-1.456666000	1.251190000
H	1.188061000	-2.047461000	1.955839000
H	2.366515000	-2.165012000	0.639421000
N	-1.307591000	-2.747681000	-0.768864000
C	-0.909940000	-1.731890000	0.061015000
C	2.291699000	2.367977000	-1.918121000
H	1.206034000	2.425815000	-1.762601000
C	2.746242000	-0.530653000	2.030712000
C	4.046409000	2.184460000	-0.056851000
H	4.678610000	2.972271000	-0.441355000
C	2.031779000	0.139688000	3.213901000
H	1.726641000	-0.618062000	3.946233000
H	1.135945000	0.669847000	2.865144000
H	2.691170000	0.858790000	3.717712000
C	-2.691579000	-2.801377000	0.958992000
H	-3.459632000	-3.016854000	1.689379000
C	-2.406497000	-3.405580000	-0.225258000

H	-2.889178000	-4.244842000	-0.702303000
C	4.219765000	1.366921000	1.024536000
H	5.023539000	1.321029000	1.746785000
C	3.996749000	-1.280445000	2.498542000
H	4.659806000	-0.643405000	3.100278000
H	4.562744000	-1.673696000	1.644346000
H	3.693260000	-2.125799000	3.128266000
C	-0.704212000	-3.069672000	-2.111015000
C	-1.727253000	-0.875354000	2.288549000
H	-0.923463000	-0.166229000	2.044593000
C	-1.358109000	-1.652465000	3.552053000
H	-2.143922000	-2.371545000	3.821436000
H	-1.238385000	-0.957444000	4.392435000
H	-0.418588000	-2.203069000	3.421415000
C	2.815661000	3.771374000	-2.206891000
H	3.885754000	3.764832000	-2.454785000
H	2.652328000	4.450250000	-1.360005000
H	2.283969000	4.177886000	-3.075283000
C	2.581885000	1.384095000	-3.053452000
H	2.130249000	1.742209000	-3.987188000
H	2.168641000	0.393838000	-2.821675000
H	3.664454000	1.282718000	-3.209633000
C	-1.101754000	-1.961604000	-3.097477000
H	-2.195047000	-1.885850000	-3.175892000
H	-0.706803000	-2.190124000	-4.095370000
H	-0.697112000	-0.991747000	-2.787391000
C	-3.042477000	-0.111028000	2.430503000
H	-3.290076000	0.433301000	1.512767000
H	-2.955759000	0.617495000	3.245979000
H	-3.874749000	-0.784960000	2.676354000
C	-1.249582000	-4.416524000	-2.604362000
H	-1.032926000	-5.227133000	-1.896512000
H	-0.757703000	-4.661329000	-3.552959000
H	-2.329262000	-4.384793000	-2.799469000
C	0.820541000	-3.169657000	-1.976167000
H	1.258161000	-2.202889000	-1.686345000
H	1.252202000	-3.467236000	-2.939706000
H	1.099147000	-3.915221000	-1.220629000
N	-0.953127000	2.392354000	-0.054286000
N	-2.341287000	0.851189000	-1.013988000
H	-0.176350000	2.886030000	0.365530000
C	-2.261924000	2.864035000	0.046540000
B	-0.962371000	1.092020000	-0.678613000
C	-3.121609000	1.914222000	-0.562772000
C	-2.774087000	4.026768000	0.622464000
H	-2.115899000	4.757262000	1.092774000
H	-2.748213000	0.054946000	-1.486882000
C	-4.500924000	2.120452000	-0.606739000
H	-5.162359000	1.390938000	-1.073964000
C	-4.156579000	4.227910000	0.574428000

H	-4.580169000	5.130018000	1.013711000
C	-5.007124000	3.289685000	-0.031538000
H	-6.080079000	3.475304000	-0.055034000
H	-0.032161000	0.570952000	-1.430271000

Complex 8b[7]

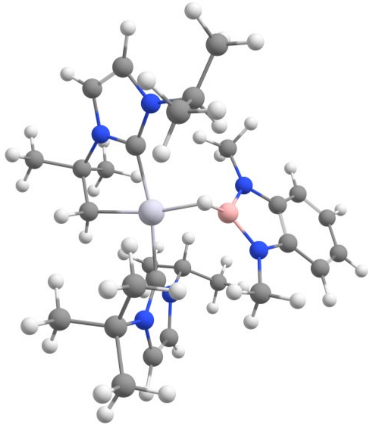

Pt	-0.720274000	0.288515000	-0.058172000
N	-3.143549000	-0.896946000	0.996317000
N	-2.731836000	-2.050919000	-0.788365000
N	1.654052000	1.822986000	1.076641000
C	-2.255674000	-1.026510000	-0.025554000
C	-2.005689000	1.184096000	1.318462000
H	-1.484952000	1.812151000	2.051903000
H	-2.677148000	1.839095000	0.741087000
N	0.781753000	3.003866000	-0.522781000
C	0.651507000	1.813844000	0.149707000
C	-2.095686000	-2.485299000	-2.054899000
H	-1.015501000	-2.450900000	-1.855919000
C	-2.854509000	0.115464000	2.044096000
C	-3.905805000	-2.549045000	-0.238032000
H	-4.461902000	-3.362526000	-0.682282000
C	-2.056395000	-0.569655000	3.163667000
H	-1.839917000	0.148693000	3.964132000
H	-1.105440000	-0.952824000	2.771057000
H	-2.623660000	-1.404130000	3.596822000
C	2.392579000	2.989810000	0.998172000
H	3.238109000	3.193626000	1.640512000
C	1.846579000	3.731127000	-0.000867000
H	2.139838000	4.699484000	-0.376554000
C	-4.159451000	-1.825322000	0.893361000
H	-4.969981000	-1.899095000	1.605705000
C	-4.162359000	0.702359000	2.581957000
H	-4.747239000	-0.039847000	3.143000000
H	-4.781755000	1.098430000	1.767051000
H	-3.930753000	1.525170000	3.269395000
C	-0.165244000	3.579965000	-1.546144000
C	1.885480000	0.764277000	2.088015000
H	1.332831000	-0.105237000	1.707036000

C	1.290927000	1.193056000	3.429840000
H	1.802419000	2.084611000	3.817914000
H	1.407187000	0.387275000	4.165519000
H	0.223279000	1.419417000	3.330928000
C	-2.475630000	-3.919949000	-2.407183000
H	-3.532463000	-4.003947000	-2.694738000
H	-2.277885000	-4.607753000	-1.575046000
H	-1.878227000	-4.245570000	-3.267027000
C	-2.430791000	-1.491702000	-3.168540000
H	-1.909835000	-1.771641000	-4.092864000
H	-2.121996000	-0.476874000	-2.887254000
H	-3.510816000	-1.485222000	-3.368510000
C	-0.680905000	2.495097000	-2.497032000
H	0.133458000	2.040649000	-3.074479000
H	-1.385730000	2.950851000	-3.203287000
H	-1.208549000	1.697060000	-1.950982000
C	3.368134000	0.412457000	2.191858000
H	3.802641000	0.203599000	1.208288000
H	3.483153000	-0.490907000	2.802950000
H	3.944286000	1.212990000	2.676069000
C	0.574338000	4.640946000	-2.375810000
H	0.826343000	5.534544000	-1.791412000
H	-0.084199000	4.968179000	-3.189022000
H	1.490465000	4.234579000	-2.824299000
C	-1.330644000	4.224364000	-0.780783000
H	-1.878747000	3.469513000	-0.206799000
H	-2.025746000	4.700287000	-1.484078000
H	-0.962692000	4.990901000	-0.086383000
N	1.289574000	-2.219240000	-0.017869000
N	2.357896000	-0.540376000	-1.194710000
C	0.385258000	-3.098598000	0.699140000
H	0.438234000	-4.122602000	0.301549000
H	-0.638675000	-2.729940000	0.607798000
H	0.641205000	-3.133726000	1.769230000
C	2.664844000	-2.455634000	0.003191000
B	1.064740000	-0.979590000	-0.732833000
C	3.317868000	-1.434144000	-0.730379000
C	3.396269000	-3.469018000	0.624357000
H	2.902176000	-4.255747000	1.192982000
C	2.719768000	0.622331000	-1.979068000
H	1.810222000	1.104728000	-2.344747000
H	3.339033000	0.336110000	-2.840626000
H	3.278742000	1.352233000	-1.372576000
C	4.707072000	-1.421051000	-0.864697000
H	5.211808000	-0.636504000	-1.427100000
C	4.789478000	-3.447914000	0.491653000
H	5.381894000	-4.229820000	0.965015000
C	5.434383000	-2.441972000	-0.242776000
H	6.519945000	-2.454192000	-0.330977000
H	0.034755000	-0.587225000	-1.406335000

Complex 9a[8]

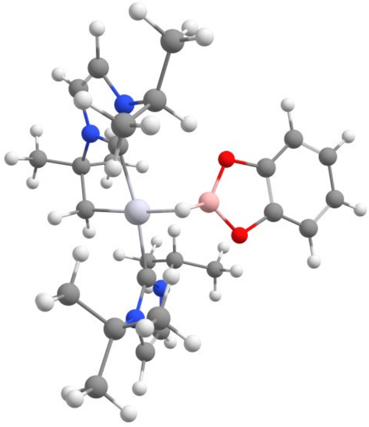

C	2.205044970	-0.562050610	0.189526950
C	4.451607750	-0.454461020	0.202991560
H	5.442005460	-0.230132450	-0.167075960
C	4.063920690	-1.033481510	1.379046450
H	4.653125650	-1.396522180	2.210102080
C	1.712620010	-1.592651940	2.348916180
C	2.281636990	-2.814921330	3.074820450
H	2.586465280	-3.591400570	2.361686490
H	1.510025530	-3.234423330	3.732147190
H	3.143263310	-2.554114840	3.704706960
C	0.457141220	-1.956321030	1.527505640
H	-0.416741920	-2.028015050	2.184278300
H	0.593727980	-2.939665110	1.048473590
C	3.230997310	0.374807790	-1.894680170
H	2.399788240	1.089628060	-1.876014450
C	4.504840180	1.130480160	-2.259043440
H	4.744269210	1.902358290	-1.516786740
H	4.358507370	1.624890920	-3.226710930
H	5.365496440	0.455959330	-2.365755870
C	2.917447100	-0.756962300	-2.875992290
H	3.726694720	-1.499764300	-2.882706070
H	2.809663350	-0.354801030	-3.891295340
H	1.982572000	-1.263127390	-2.601937070
C	-1.850424890	-0.945039010	-0.016448080
C	-3.985706420	-0.916355740	0.727139070
H	-4.813376650	-0.649021330	1.369868700
C	-3.948879640	-1.702927950	-0.380725560
H	-4.747761850	-2.241259600	-0.867342600
C	-2.175616610	-2.426642010	-2.079446310
C	-0.927634900	-3.263025830	-1.767401550
H	-0.076222010	-2.622998600	-1.489066310
H	-0.640095850	-3.835789590	-2.657582810
H	-1.117687800	-3.963396820	-0.944164660
C	-1.885600350	-1.367419980	-3.152155710
H	-2.777705530	-0.759567090	-3.349276930
H	-1.586406170	-1.858418410	-4.086864460

H	-1.074461500	-0.700126530	-2.841707750
C	-3.287115000	-3.363922790	-2.571797360
H	-3.563021800	-4.106091740	-1.811293180
H	-2.916611730	-3.906956510	-3.449098360
H	-4.184345690	-2.816201430	-2.887045900
C	-2.313691080	0.437323400	2.048883980
H	-1.256533330	0.669052980	1.860070520
C	-2.445972920	-0.280862550	3.392319940
H	-3.494134910	-0.530912170	3.605750780
H	-2.086703880	0.372220630	4.197340350
H	-1.861840570	-1.208494550	3.412710100
C	-3.132652680	1.727098920	1.999729020
H	-3.091339230	2.177312590	1.003449740
H	-2.730925030	2.446439930	2.723824240
H	-4.183743530	1.543998010	2.261972440
C	1.417437230	-0.452643260	3.335359000
H	2.328146460	-0.146444040	3.866637030
H	0.680415530	-0.781572230	4.078497460
H	1.015493770	0.418001520	2.801441020
N	-2.633792820	-1.721411270	-0.829058490
N	-2.696364740	-0.462791130	0.937175550
N	3.298701070	-0.167825690	-0.514853450
N	2.686143130	-1.098636480	1.342199530
Pt	0.192187370	-0.626938700	-0.052868500
H	0.154439730	0.369480170	-1.501227440
B	-0.080002770	1.394323030	-0.688000510
O	0.933874760	2.341206710	-0.445849800
O	-1.334730480	2.019405740	-0.793740290
C	0.275066680	3.555163210	-0.330767610
C	-1.092280880	3.361777050	-0.546856540
C	0.807394800	4.803924910	-0.051733780
C	-1.999977040	4.408203820	-0.501244700
C	-0.102258980	5.871008840	-0.000739500
H	1.873632130	4.943934500	0.115790710
C	-1.473147120	5.678104730	-0.221456380
H	-3.061706010	4.250065600	-0.680953030
H	0.268189060	6.872875440	0.211597620
H	-2.146296230	6.533130500	-0.178710750

Complex 9b[8]

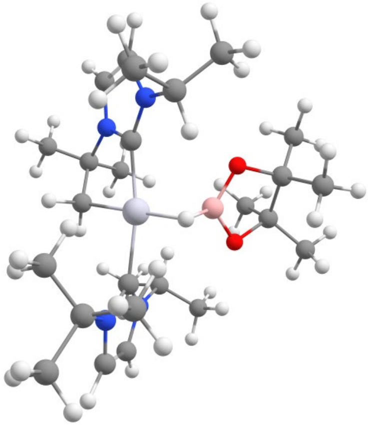

C	1.473500000	-1.490758000	0.411341000
C	3.449540000	-2.544137000	0.638652000
H	4.418897000	-2.917620000	0.337768000
C	2.786099000	-2.666190000	1.826407000
H	3.075823000	-3.155679000	2.746256000
C	0.432935000	-1.851278000	2.592095000
C	0.292382000	-3.086982000	3.485538000
H	0.221318000	-4.002403000	2.884311000
H	-0.623054000	-2.995635000	4.083139000
H	1.133345000	-3.187033000	4.185683000
C	-0.789466000	-1.676781000	1.665308000
H	-1.623649000	-1.236643000	2.224425000
H	-1.119821000	-2.660339000	1.294753000
C	2.909403000	-1.590351000	-1.652830000
H	2.153844000	-0.863908000	-1.968523000
C	4.291542000	-0.971932000	-1.844442000
H	4.387134000	-0.061231000	-1.243239000
H	4.429667000	-0.709202000	-2.900598000
H	5.094185000	-1.671118000	-1.572763000
C	2.716352000	-2.894223000	-2.430061000
H	3.444702000	-3.654574000	-2.116499000
H	2.856633000	-2.714074000	-3.503381000
H	1.706660000	-3.295296000	-2.273443000
C	-2.215970000	0.138686000	-0.190906000
C	-4.151356000	1.206182000	0.293362000
H	-4.809083000	1.890117000	0.811992000
C	-4.401860000	0.355114000	-0.735615000
H	-5.323719000	0.174522000	-1.266791000
C	-3.049706000	-1.289891000	-2.152598000
C	-2.374209000	-2.562714000	-1.625217000
H	-1.342892000	-2.360492000	-1.296291000
H	-2.331355000	-3.311688000	-2.425731000
H	-2.932557000	-2.983367000	-0.779192000
C	-2.213604000	-0.631639000	-3.259425000
H	-2.692516000	0.291295000	-3.610681000
H	-2.118518000	-1.318497000	-4.110015000
H	-1.206413000	-0.388362000	-2.903216000

C	-4.431379000	-1.660678000	-2.709923000
H	-5.081029000	-2.095384000	-1.938972000
H	-4.294778000	-2.414551000	-3.494003000
H	-4.938398000	-0.803499000	-3.171071000
C	-2.145965000	1.824776000	1.691756000
H	-1.088105000	1.544425000	1.612106000
C	-2.690515000	1.411194000	3.059455000
H	-3.748239000	1.687082000	3.169377000
H	-2.127091000	1.921457000	3.850798000
H	-2.600676000	0.329456000	3.215219000
C	-2.289037000	3.324493000	1.434184000
H	-1.879176000	3.583335000	0.452084000
H	-1.744489000	3.885398000	2.204306000
H	-3.338934000	3.643596000	1.483420000
C	0.673816000	-0.589809000	3.434591000
H	1.596102000	-0.678749000	4.023562000
H	-0.163399000	-0.435237000	4.127040000
H	0.754669000	0.290536000	2.783958000
N	-3.212538000	-0.301124000	-1.026235000
N	-2.813365000	1.061756000	0.613836000
N	2.630873000	-1.820935000	-0.218306000
N	1.574920000	-2.025375000	1.659418000
Pt	-0.273512000	-0.556056000	-0.014931000
H	0.153471000	0.297703000	-1.481561000
B	0.773756000	1.210850000	-0.716154000
O	0.190125000	2.440461000	-0.475489000
O	2.150013000	1.264196000	-0.833744000
C	1.300494000	3.335172000	-0.084240000
C	2.516403000	2.691731000	-0.839026000
C	1.435222000	3.232463000	1.436202000
C	0.954555000	4.752318000	-0.510601000
C	3.859981000	2.850211000	-0.143149000
C	2.606783000	3.113606000	-2.305746000
H	2.209448000	3.910662000	1.815209000
H	0.483349000	3.509061000	1.902643000
H	1.687423000	2.207863000	1.740186000
H	0.109259000	5.126731000	0.080076000
H	1.809462000	5.419905000	-0.338512000
H	0.680693000	4.798467000	-1.569704000
H	4.651961000	2.396979000	-0.752474000
H	4.101500000	3.914040000	-0.016564000
H	3.865493000	2.368236000	0.840111000
H	2.950302000	4.151206000	-2.401679000
H	3.326585000	2.464207000	-2.818971000
H	1.635166000	3.017444000	-2.806975000

Complex 10[9]

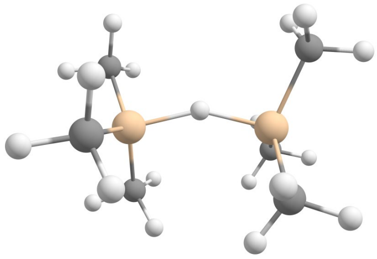

Si	-1.588156480	-0.002615300	0.048466310
C	-1.885246480	-1.793007150	-0.344252060
H	-1.330493200	-2.114338730	-1.234838410
H	-2.957368230	-1.933471000	-0.551869440
H	-1.622602270	-2.445418260	0.498259070
C	-2.283779210	0.620287090	1.653705130
H	-3.380178960	0.670079570	1.562881840
H	-1.922812240	1.629115590	1.890720390
H	-2.047921120	-0.051177960	2.488904930
C	-1.676110670	1.170678980	-1.389480550
H	-1.346710770	2.180716010	-1.114396870
H	-2.724398280	1.241606440	-1.718675430
H	-1.086956710	0.820387720	-2.246909140
Si	1.587986620	0.002184650	0.049066560
H	-0.000116640	-0.001731070	0.432911210
C	1.674299110	-1.133424980	-1.418857680
H	2.722982230	-1.197773780	-1.748439600
H	1.087126610	-0.760129020	-2.267983570
H	1.342835820	-2.149669900	-1.170293460
C	2.280887570	-0.665020910	1.637618460
H	1.915694040	-1.678196250	1.848418900
H	2.047851890	-0.014583140	2.490054040
H	3.377018610	-0.717031650	1.545452630
C	1.890088780	1.801216680	-0.297164600
H	1.632724380	2.432075810	0.563169270
H	1.333056840	2.147577320	-1.176891310
H	2.961811400	1.943613150	-0.505347350

Complex 11a

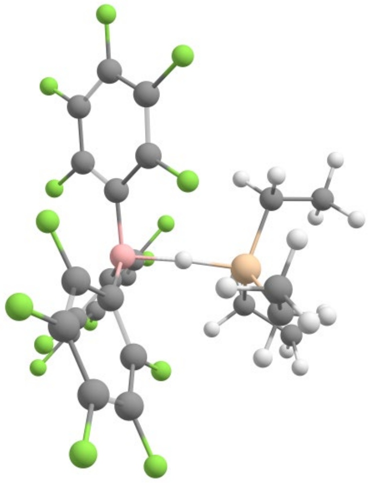

B	0.017334000	0.040159000	-0.439988000
F	-0.852122000	-2.556359000	0.474720000
F	0.298831000	-2.255748000	-2.341806000
F	-3.301182000	-3.630654000	0.146918000
F	-2.200639000	1.243353000	-2.068636000
F	2.558636000	0.438151000	0.874336000
F	-4.627793000	0.151592000	-2.386911000
F	1.997485000	1.449439000	-2.173323000
F	-1.611760000	2.052643000	0.876864000
F	-5.215041000	-2.283999000	-1.286105000
F	2.527567000	-3.714283000	-2.667945000
F	4.786999000	-3.142583000	-1.232610000
F	2.293443000	4.108583000	-2.329003000
F	4.781984000	-1.052255000	0.545244000
F	-1.274299000	4.722281000	0.734655000
F	0.678959000	5.777845000	-0.879033000
C	-1.765562000	-1.852293000	-0.242940000
C	-1.418205000	-0.595929000	-0.755033000
C	-2.423775000	0.044891000	-1.488422000
C	1.318057000	-0.859145000	-0.691297000
C	1.373817000	-1.928880000	-1.592019000
C	-3.999168000	-1.747338000	-1.119979000
C	0.202579000	1.623898000	-0.600875000
C	-3.020940000	-2.432759000	-0.394486000
C	2.510323000	-0.594200000	-0.007890000
C	-3.696015000	-0.503611000	-1.675934000
C	1.175917000	2.211747000	-1.418562000
C	2.522918000	-2.699500000	-1.788193000
C	3.674401000	-1.342314000	-0.159153000
C	1.347218000	3.594596000	-1.526175000
C	-0.613304000	2.526378000	0.089868000
C	0.524260000	4.449949000	-0.791854000
C	-0.469354000	3.909670000	0.028293000
C	3.679366000	-2.408384000	-1.062415000
Si	-0.071191000	-0.252520000	2.606538000
C	-1.791549000	-0.866906000	3.048855000

H	-1.888874000	-1.892863000	2.663426000
H	-1.775861000	-0.970041000	4.148989000
C	0.353154000	1.446329000	3.284198000
H	1.161949000	1.861897000	2.664572000
H	-0.514201000	2.107329000	3.145444000
C	-2.988713000	-0.006070000	2.615960000
H	-2.909548000	1.022914000	2.990266000
H	-3.930051000	-0.426627000	2.995176000
H	-3.063526000	0.051990000	1.523125000
C	1.244353000	-1.560777000	2.915230000
H	1.250184000	-2.236661000	2.046776000
H	2.226188000	-1.065283000	2.925574000
C	0.776252000	1.403991000	4.766088000
H	-0.018372000	0.995887000	5.407152000
H	1.009317000	2.411349000	5.137770000
H	1.672017000	0.785040000	4.914244000
C	1.027346000	-2.365126000	4.212255000
H	1.016602000	-1.717703000	5.100286000
H	1.829548000	-3.102420000	4.354010000
H	0.076627000	-2.914691000	4.192986000
H	-0.061852000	-0.036973000	1.039192000

Complex 11b[10]

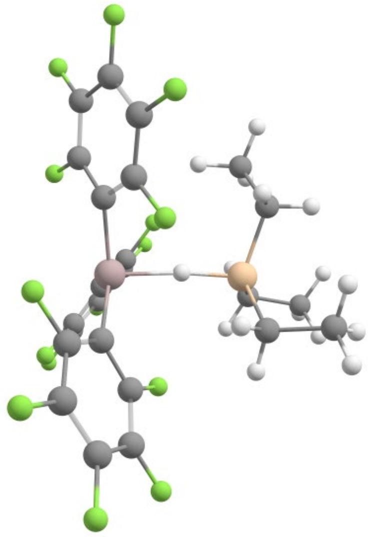

Al	0.014367000	0.044993000	-0.666317000
F	-0.208899000	-2.854731000	0.324555000
F	1.384558000	-2.289924000	-2.396254000
F	-2.279772000	-4.619202000	0.308442000
F	-2.826600000	0.230172000	-2.174007000
F	2.557758000	1.144172000	0.674224000
F	-4.890874000	-1.539691000	-2.182865000
F	1.486238000	2.430787000	-2.192859000
F	-2.266601000	1.701585000	0.633505000
F	-4.622001000	-3.959700000	-0.943964000
F	3.973144000	-3.127049000	-2.440543000

F	5.852297000	-1.836855000	-0.934314000
F	0.985736000	5.102455000	-2.158416000
F	5.145858000	0.297954000	0.626817000
F	-2.753189000	4.377448000	0.671942000
F	-1.129783000	6.078496000	-0.729562000
C	-1.355117000	-2.509310000	-0.328005000
C	-1.444519000	-1.259343000	-0.937053000
C	-2.656186000	-0.965296000	-1.558672000
C	1.888775000	-0.543311000	-0.862906000
C	2.293070000	-1.627130000	-1.638936000
C	-3.600512000	-3.092866000	-0.942077000
C	-0.373694000	1.976908000	-0.779872000
C	-2.399847000	-3.430767000	-0.308853000
C	2.885288000	0.084589000	-0.120009000
C	-3.734694000	-1.852849000	-1.574847000
C	0.427870000	2.881195000	-1.474425000
C	3.614027000	-2.078963000	-1.680511000
C	4.216800000	-0.325961000	-0.118761000
C	0.195277000	4.258152000	-1.474668000
C	-1.443346000	2.523649000	-0.075252000
C	-0.887381000	4.760884000	-0.745799000
C	-1.718058000	3.889336000	-0.033885000
C	4.578964000	-1.420062000	-0.911653000
Si	0.034114000	-0.239351000	2.642656000
C	-1.433533000	-1.323718000	3.086780000
H	-1.230638000	-2.330428000	2.689048000
H	-1.433603000	-1.432161000	4.184564000
C	-0.096629000	1.537647000	3.230753000
H	0.674042000	2.120726000	2.703086000
H	-1.066083000	1.936185000	2.895505000
C	-2.798670000	-0.811487000	2.596223000
H	-3.023085000	0.190414000	2.985980000
H	-3.609751000	-1.480671000	2.914234000
H	-2.830703000	-0.746777000	1.500682000
C	1.688850000	-1.073405000	2.958970000
H	1.842387000	-1.807419000	2.152237000
H	2.482499000	-0.320421000	2.841113000
C	0.056543000	1.693413000	4.755915000
H	-0.697287000	1.109713000	5.303548000
H	-0.058871000	2.742575000	5.060836000
H	1.045827000	1.360513000	5.099499000
C	1.789956000	-1.764787000	4.332548000
H	1.649333000	-1.056244000	5.160761000
H	2.774672000	-2.233103000	4.466442000
H	1.033657000	-2.553909000	4.443453000
H	-0.047010000	-0.115356000	1.113615000

Complex 12[11]

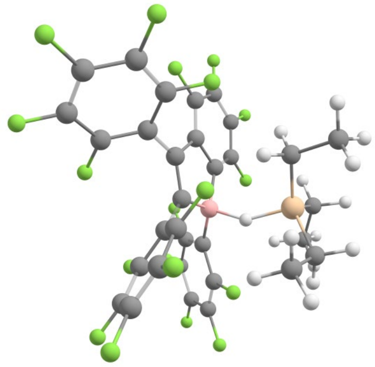

F	0.908702000	-2.352365000	-1.779278000
F	2.897248000	3.132607000	-0.151643000
F	-2.486328000	3.403017000	-1.194174000
F	-0.971956000	5.637091000	-1.190266000
F	-2.129732000	-1.173177000	1.691802000
F	1.704864000	5.518554000	-0.673833000
F	-3.237247000	1.819952000	1.095093000
F	-2.640198000	-3.784732000	2.121253000
F	2.626110000	1.253455000	-2.491032000
F	-1.364445000	-5.706373000	0.638560000
F	-2.173775000	-0.976778000	-2.595379000
F	-5.875177000	1.288454000	0.732650000
C	-2.623561000	0.452683000	-0.745413000
F	0.416872000	-4.966840000	-1.305866000
F	2.624657000	-1.758010000	1.198086000
C	-1.186598000	0.718684000	-0.524267000
C	0.811184000	1.996575000	-0.326041000
C	1.560230000	3.152810000	-0.367954000
C	-3.605721000	1.012981000	0.080855000
F	4.932445000	-2.837444000	0.305036000
C	-1.175223000	3.286330000	-0.892269000
C	-1.115475000	-4.409555000	0.413825000
C	0.054570000	-2.675248000	-0.790875000
F	-4.805899000	-1.518455000	-2.937645000
F	4.927771000	0.164431000	-3.366783000
C	-0.399965000	4.456832000	-0.908248000
C	-1.500688000	-2.083374000	0.914624000
C	-0.251817000	-0.241528000	-0.252991000
C	0.968744000	4.395989000	-0.645828000
C	-0.583720000	2.064693000	-0.587623000
F	6.102792000	-1.886601000	-1.986158000
F	-6.669079000	-0.387174000	-1.282379000
C	-0.198987000	-4.029200000	-0.570368000
C	-0.574877000	-1.659996000	-0.052836000
C	-4.962179000	0.742933000	-0.085432000
C	-5.368739000	-0.117898000	-1.109552000
C	4.362729000	-0.292536000	-2.237621000

C	-3.062629000	-0.411026000	-1.758083000
B	1.183884000	0.464783000	-0.070677000
C	3.175984000	-1.244288000	0.066012000
C	2.547637000	-0.179970000	-0.586121000
C	4.963759000	-1.341092000	-1.537923000
C	3.175118000	0.263023000	-1.756003000
C	4.364814000	-1.824078000	-0.371728000
C	-1.772116000	-3.428595000	1.160317000
C	-4.415657000	-0.697668000	-1.950458000
Si	1.266802000	0.940842000	2.771840000
C	1.489480000	-0.569766000	3.862901000
H	2.507448000	-0.955870000	3.709551000
H	1.462457000	-0.182107000	4.897199000
C	2.716635000	2.121737000	2.864940000
H	2.480213000	3.019003000	2.276435000
H	2.770398000	2.454392000	3.916579000
C	-0.435951000	1.706570000	2.868577000
H	-0.442861000	2.622998000	2.260835000
H	-1.143823000	1.020300000	2.382231000
C	0.452790000	-1.687238000	3.677041000
H	-0.575339000	-1.317103000	3.785049000
H	0.596466000	-2.489395000	4.413399000
H	0.540042000	-2.135439000	2.679266000
C	-0.884166000	1.999923000	4.313833000
H	-0.922695000	1.084125000	4.920136000
H	-1.889402000	2.442054000	4.325049000
H	-0.209986000	2.705037000	4.820695000
C	4.061116000	1.521544000	2.417421000
H	4.323344000	0.625263000	2.996272000
H	4.876982000	2.245947000	2.541698000
H	4.030423000	1.238788000	1.357558000
H	1.332727000	0.298275000	1.317006000

Complex 13[12]

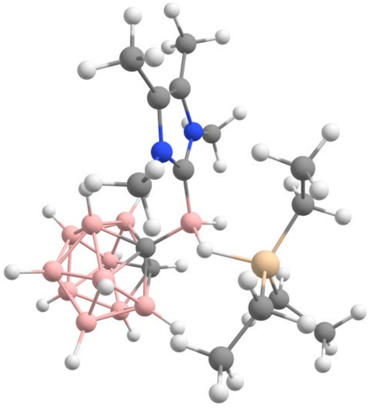

N	-1.864089580	-0.970686760	0.996035390
N	-2.084877230	-1.296791570	-1.128312760
B	0.026932630	0.147989760	-0.498121770
H	0.037087720	0.597969930	-1.617124370
C	1.404352890	-0.618654440	-0.147371490

C	2.600941240	-0.586387520	-1.298584030
H	2.371031830	0.006949910	-2.179490040
B	2.841633270	0.297137460	0.140540430
H	2.752525900	1.480001750	0.107871500
B	2.122083150	-0.718170120	1.400418700
H	1.567202370	-0.170021290	2.300452970
B	1.414462290	-2.148066980	0.629226240
H	0.366814730	-2.581291660	0.995449670
B	1.684732360	-2.018644220	-1.119291970
H	0.871961080	-2.275478510	-1.948677930
B	3.444510420	-2.056622910	-1.409688480
H	3.816256680	-2.403555750	-2.483855590
B	4.159775310	-0.620560980	-0.628548750
H	5.013037970	-0.001426670	-1.177081070
B	3.880300590	-0.741217850	1.128951730
H	4.629233040	-0.249795230	1.912630020
B	2.995266700	-2.269953170	1.432602430
H	3.112770620	-2.879582960	2.448736120
B	2.716544590	-3.080172030	-0.142723980
H	2.635777650	-4.261084020	-0.269767970
B	4.255348440	-2.211195430	0.169882730
H	5.295075180	-2.781363070	0.267212620
C	-1.303706490	-0.679117260	-0.205368110
C	-3.126962970	-1.986194400	-0.513023630
C	-2.987870430	-1.776164270	0.837765580
C	-1.366815220	-0.544027720	2.299271190
H	-0.567878390	0.187427040	2.167586430
H	-0.957306490	-1.403479760	2.842858570
H	-2.182664630	-0.093849340	2.876025390
C	-1.872293310	-1.304489700	-2.573086780
H	-1.046305260	-0.635672140	-2.820140990
H	-2.782830860	-0.968041290	-3.081877210
H	-1.622389330	-2.318093690	-2.907426000
C	-4.127903030	-2.773890870	-1.279726340
H	-4.839418960	-3.250339530	-0.597741810
H	-3.648689840	-3.566753730	-1.871455320
H	-4.701171750	-2.141049960	-1.972198190
C	-3.794432300	-2.257651560	1.989941940
H	-4.604355380	-2.907032380	1.642538020
H	-4.249638680	-1.424237550	2.544527850
H	-3.181330270	-2.833923430	2.697117160
Si	-0.419129390	2.624619320	-0.176044920
H	0.022079370	1.124920740	0.366671100
C	-2.178868610	2.539725500	-0.814378940
H	-2.350079270	3.534457740	-1.263231310
H	-2.212018080	1.835287290	-1.659223270
C	-3.282513160	2.232272890	0.210837430
H	-3.169337210	1.228730110	0.641304660
H	-4.274797600	2.277635730	-0.256475340
H	-3.279269030	2.951901350	1.039795290

C	-0.252187830	3.326787730	1.553573800
H	-0.589000740	4.376147510	1.482149080
H	-0.982584520	2.829869420	2.210912700
C	1.166451170	3.250545130	2.144399200
H	1.502375330	2.211132900	2.258729430
H	1.204930930	3.723229150	3.133932530
H	1.900655590	3.760462800	1.506682190
C	0.821390320	3.293158780	-1.402220900
H	1.821351870	2.913702990	-1.150435410
H	0.577557960	2.896208530	-2.399028040
C	0.814833930	4.837142370	-1.411228760
H	-0.174706830	5.246857660	-1.658649860
H	1.519816520	5.218992940	-2.160666720
H	1.114364140	5.250918740	-0.439103790

3. References

[1] P. Ríos, H. Fouilloux, P. Vidossich, J. Díez, A. Lledós, S. Conejero, *Angew. Chem. Int. Ed.,* **2018**, *57*, 3217-3221.
[2] J. Yang, P. S. White, C. K. Schauer, M. Brookhart, *Angew. Chem. Int. Ed.* **2008**, *47*, 4141–4143.
[3] S.T.N. Freeman, F. R. Lemke, L. Brammer, *Organometallics,* **2002**, *21*, 2030-2032.
[4] S. K. Ignatov, N. H. Rees, B. R. Tyrrell, S. R. Dubberley, A. G. Razuvaev, P. Mountford, G. I. Nikonov, *Chem. Eur. J.,* **2004**, *10*, 4991 – 4999.
[5] P. Meixner, K. Batke, A. Fischer, D. Schmitz, G. Eickerling, M. Kalter, K. Ruhland, K. Eichele, J. E. Barquera-Lozada, N. P. M. Casati, F. Montisci, P. Macchi, W. Scherer, *J. Phys. Chem. A*, **2017**, *121*, 7219−7235.
[6] L. Rocchigiani, P. H. M. Budzelaar, M. Bochmann, *Chem. Sci.* **2019**, *10*, 2633-2642.
[7] P. Ríos, F. J. Fernández-de-Córdova, J. Borge, N. Curado, A. Lledós, S. Conejero, *Eur. J. Inorg. Chem.,* **2021**, 3528-3539.
[8] P. Ríos, R. Martín-de la Calle, P. Vidossich, F. J. Fernández-de-Córdova, A. Lledós, S. Conejero, *Chem. Sci.* **2021**, *12*, 1647-1655.
[9] S. P. Hoffmann, T. Kato, F. S. Tham, C. A. Reed, *Chem. Commun.* **2006**, 767–769.
[10] J. Chen, E. Y.-X. Chen, *Angew. Chem. Int. Ed.* **2015**, *54*, 6842–6846.
[11] A. Y. Houghton, J. Hurmalainen, A. Mansikkamäki, W. E. Piers, H. M. Tuononen, *Nat. Chem.* **2014**, *6*, 983–988.
[12] Y. Liu, B. Su, W. Dong, Z. H. Li, H. Wang, *J. Am. Chem. Soc.* **2019**, *141*, 8358–8363.
